# Determinants of cervical cancer screening utilisation among women in the least developed countries: A systematic review and meta-analysis

**DOI:** 10.1371/journal.pone.0321627

**Published:** 2025-06-24

**Authors:** Tika Rana, Dorothy Ngo Sheung Chan, Bernard Man Hin Law, Kai Chow Choi, Sunil Shrestha, Winnie Kwok Wei So

**Affiliations:** 1 The Nethersole School of Nursing, Faculty of Medicine, The Chinese University of Hong Kong, Hong Kong SAR, China,; 2 Department of Research and Academics, Kathmandu Cancer Center, Tathali, Bhaktapur, Bagmati Province, Nepal,; 3 Person-Centred Research, Eastern Health Clinical School, Faculty of Medicine, Nursing andHealth Sciences, Monash University, Australia; University of Ilorin, NIGERIA

## Abstract

**Background and aims:**

Globally, cervical cancer is the fourth most common cancer among women, and more than 90% of all cervical cancer-related deaths worldwide are recorded in resource-limited countries. The present review investigated the uptake rate of cervical cancer screening and identified the factors associated with screening service utilisation in the female populations of least developed countries (LDCs).

**Methods:**

Five electronic databases (EMBASE, Ovid MEDLINE, CINAHL, Cochrane Library, and PubMed) and grey literature were searched for relevant literature using the keywords of the included studies. Both qualitative and quantitative studies were included. Three reviewers performed critical appraisals using the Mixed Methods Appraisal Tool. Meta-analysis was performed to pool the quantitative results from comparable studies.

**Results:**

A total of twenty-five studies were included in the review. The cervical cancer screening uptake rate in LDCs ranged from 4% to 21%. Multiple factors were associated with screening service utilisation among women in the LDCs, namely socio-demographic characteristics, including employment status (odds ratio (OR): 2.72; 95% CI: 1.97–3.76; *p *< 0.001); knowledge of cervical cancer and its screening (OR: 3.39; 95% CI: 2.00–5.75; *p *< 0.001); sexual and reproductive health-related factors such as parity status (OR: 2.73; 95% CI: 1.61–4.64; *p *= 0.002); healthcare providers’ recommendations (OR: 5.32; 95% CI: 2.44–11.58; *p* < 0.001); perceived risk of developing cervical cancer (OR: 3.76; 95% CI: 2.62–5.38; *p* < 0.001); use of media for cervical cancer screening promotion, including radio; cultural factors; and myths and misconceptions about cervical cancer and its screening.

**Conclusions:**

The uptake of cervical cancer screening among eligible women in the LDCs was notably low. The governments of these countries are advised to invest and allocate additional resources to advance policies and develop cervical cancer prevention programmes that are accessible, affordable, and acceptable.

## Introduction

In 2022, cervical cancer was the fourth most common cancer in women, with an estimated 660,000 newly diagnosed cases and 350,000 deaths globally, representing 6.6% of all female cancers [1,2]. More than 90% of all cervical cancer-related deaths worldwide are recorded in low- and middle-income countries (LMICs) [3–9]. It is estimated that the incidence of global cervical cancer will increase by 44.4 million cases between 2020 and 2069, nearly two-thirds of which will be diagnosed in least-developed countries (LDCs) [10]. The United Nations defines LDCs as low-income countries that face considerable structural impediments to sustainable development; the criteria for being defined as an LDC include a gross national income per capita of USD 1,088 or below, a human assets index of 60 or below, and an economic and environmental vulnerability index of 36 or above [11]. As of 2024, 44 countries were classified as LDCs worldwide [11]. In 2020, the World Health Organisation (WHO) launched a global strategy to eliminate cervical cancer as a public health problem by reducing the number of new cases per year to four or fewer per 100,000 and set three targets: 90% coverage of human papillomavirus (HPV) vaccination for girls (by 15 years of age); 70% coverage of cervical cancer screening (screening of 70% of women by 35 and 45 years of age using high-performance tests); treatment of 90% of precancerous lesions; and management of 90% of invasive cancer cases by 2030 [6,12,13]. Evidence indicates that nearly 80% of cervical cancer cases can be prevented and detected early with routine screening and early treatment [14,15]. Primarily, cervical cancer can be prevented by adopting healthy lifestyles and vaccinating with HPV among girls aged 9–14 [5]. Further, various types of screening tests, including HPV-based testing, conventional cytology (Papanicolaou [Pap] test), liquid-based cytology, and Visual Inspection with Acetic acid (VIA), can detect any precancerous changes in the cervix or early-stage cancer in adult women [5,16]. According to the WHO, HPV-based screening is superior to the Pap test or VIA in terms of the early detection and prevention of cervical cancer [7,17]. The HPV-based screening test detects the high-risk strains of HPV that are responsible for causing almost all cervical cancer cases and helps save more women’s lives than the Pap test or VIA [17]. It is a cost-effective screen-and-treat approach, and women aged 30 years or above are strongly advised to take regular HPV-based screening tests every 5–10 years [15]. The utilisation of self-sampling methods is also encouraged and can increase adherence among under-screened women, with acceptance rates ranging from 90.5% to 97.3% [18]. However, HPV-based testing programs are not yet operational in many countries, particularly in LDCs, due to suboptimal resources and the unavailability of affordable and clinically validated HPV tests, where VIA is recommended as a primary screening test [7,13,17,19]. The VIA involves an inspection of the cervix after applying 3%–5% acetic acid (vinegar) directly to the cervix to detect any abnormalities in it [20]. After the application of vinegar, abnormal cells in the cervix temporarily turn white (aceto-white), indicating the need for further assessments [20]. Most importantly, this screening provides instant results, allowing women to undergo screening and treatment in a single visit if any abnormalities have been detected [5,14,21]. The VIA method has an average sensitivity of 77% and an average specificity of 86% to detect precancerous and cancerous cells of the cervix [22].

Despite the wide availability of these effective cervical cancer screening tests, some screening tests, such as the Pap test, are not commonly available in LDCs due to a lack of resources, the sub-optimal performance of cytology, lack of quality control, and inefficient processes of investigation and treatment in healthcare systems; as a result, the uptake of cervical cancer screening in LDCs is significantly lower than that in developed countries [7,23]. The WHO estimated that only around 5% of the women in LDCs have been screened for cervical cancer compared with 60% in high-income countries [20,23]. For example, only 1%, 6%, 12%, and 16% of age-eligible women in Ethiopia, South Africa, Bhutan, and Nepal, respectively, have undergone cervical cancer screening [24–26].

Several reviews have identified that cervical cancer screening utilisation is associated with various factors. For example, Yimer et al. (2021) found that cervical cancer screening utilisation was positively associated with knowledge of the disease but negatively associated with a lack of formal education. Bruni et al. (2022) and Bogdanova et al. (2022) identified being older, being diagnosed with a sexually transmitted disease, fear of a negative result after the screening, the lack of a national screening programme that is accessible to women, inadequate training for healthcare providers, and stigma associated with the screening procedures as other factors associated with cervical cancer screening utilisation [7,19]. The absence of a routine screening programme for cervical cancer and a lack of official recommendations for screening also discourage screening service utilisation among women. More than 60% of low-income countries and almost 45% of LMICs still lack official recommendations for cervical cancer screening [7].

In LDCs, understanding the coverage of and factors associated with the uptake of cervical cancer screening is essential to address the barriers to implementing effective interventions and thereby reducing the cervical cancer burden. Although previous systematic reviews and meta-analyses have explored the barriers to and factors associated with cervical cancer screening utilisation in LMICs, sub-Saharan Africa, and Ethiopia, none have focused on LDCs [8,9,27]. Thus, our systematic review and meta-analysis aimed to examine the cervical cancer screening uptake rate and identify its associated factors among women in LDCs.

## Methods

The review followed the Preferred Reporting Items for Systematic Reviews and Meta-analysis (PRISMA) guidelines, and the relevant parts are reported [28]. The review protocol has been registered in the International Prospective Register of Systematic Reviews (PROSPERO; registration number **CRD42022330199**).

### Search strategy

A literature search was conducted in December 2023 across five databases, EMBASE, MEDLINE (Ovid), CINAHL, Cochrane Library and PubMed, and grey literature. The relevant studies were searched using combinations of keywords, such as ‘cervical cancer, screening or prevention or early detection of cancer or cervical smear or Pap smear test or Pap test or Visual Inspection with Acetic Acid or Papanicolaou test’, ‘factor or predictor, least developed countries or low-income countries and ‘women or female’. Various Medical Subject Headings, such as ‘uterine cervical neoplasms’, ‘early detection of cancer’, ‘poor’ and ‘women’, were also used to search for relevant studies. Regarding the types of screening test, both the Pap test and VIA screening are used in the LDCs; thus, both were used as key terms to identify relevant studies [11]. The reference lists of the retrieved studies were also searched to identify further relevant studies. Studies conducted in countries other than LDCs and published in languages other than English were excluded [11]. To include all relevant studies conducted in the LDCs, no restriction was placed on the year of publication [29] (see S1 Table).

### Study selection

Initially, the identified studies were imported into Endnote, and duplicate studies were removed [30]. The three reviewers independently screened the studies based on their abstracts and titles. The full texts of the studies were then systematically reviewed and assessed for eligibility against the inclusion and exclusion criteria. Any disagreements were resolved through discussion until a consensus was reached.

## Data extraction

Both qualitative and quantitative studies that examined cervical cancer screening uptake and its associated factors in LDCs were included in the review. Two reviewers extracted and recorded the key characteristics of the included studies, namely the lead author name, study year, country, design, population, sample size, cervical cancer screening uptake rate, and factors associated with cervical cancer screening utilisation, in a Microsoft Excel sheet. Discrepancies were resolved through discussion until a consensus was reached.

### Quality assessment of included studies

To assess the quality of the studies, the Mixed Methods Appraisal Tool (MMAT) version 2018 was used [31]. This tool is used to appraise the quality of primary research studies based on experiment or observation, and it has been validated for its content and tested for its reliability [32]. This assessment tool allows for appraisal of the methodological quality of studies with different designs, including quantitative, qualitative, and mixed-method studies. Every criterion is rated on a categorical scale of ‘yes’, ‘no’, and ‘cannot tell’. Based on the suggestions made in the previous version of the MMAT, the overall quality score for the methodological items can be presented using descriptors such as stars (*) or % (5***** or 100% quality criteria met, 4**** or 80% quality criteria met, 3*** or 60% quality criteria met, 2** or 40% quality criteria met and 1* or 20% quality criteria met) [33]. The critical appraisal was performed by three independent reviewers, and any disagreement was resolved through discussion and consensus.

### Data synthesis

We combined the quantitative and qualitative findings to present a comprehensive synthesis of the evidence. The results of quantitative studies included in the review were pooled for meta-analysis if appropriate. Meta-analysis was conducted using the Cochrane Collaboration’s Review Manager (RevMan 5.4.1) [34]. The generic inverse variance method was used to pool the results of individual studies with effect size estimates, whose standard errors were reported or could be estimated from other reported parameters. The standard error is needed to calculate the inverse variance weight, which is used to compute the pooled effect estimate and its 95% confidence interval (CI). In view of the heterogeneity of the study populations, a random-effects model was used for all of the meta-analysis [35]. Odds ratios (ORs) and 95% CIs were used to summarise the effect sizes of associated factors for cervical cancer screening uptake of the included studies. Heterogeneity among studies was assessed using I^2^ with *I*^2 ^< 25%, 25 ≤ *I*^2 ^< 50%, and *I*^2 ^≥ 50%, categorising heterogeneity as low, medium, and high, respectively [35]. Sensitivity analysis was used to identify the reasons for the heterogeneity and evaluate the robustness of the results by excluding the studies one by one [36]. The qualitative data were synthesised narratively to ensure empirical faithfulness to the original data and allow their integration with the quantitative data [37].

## Results

### Search results

**Fig 1:**
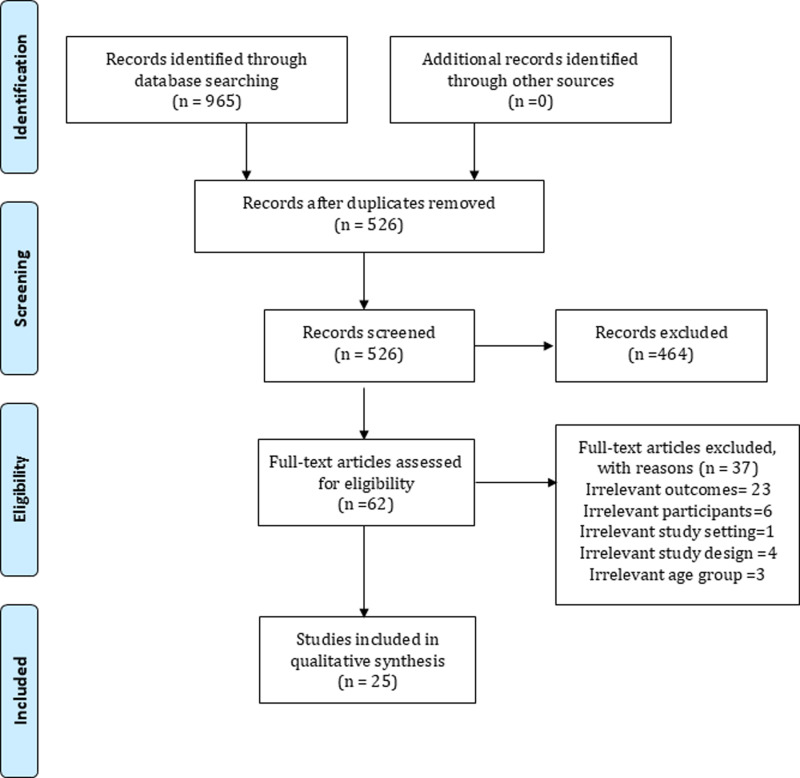
Study selection flow diagram.

A total of 965 articles were retrieved from the five databases, the grey literature, and the manual search of the reference lists of the retrieved articles. Among them, 439 articles were found to be duplicates and hence removed. After screening the titles and abstracts of the remaining 526 articles, 62 relevant articles were found, and their full texts were reviewed. A total of 37 articles were further removed after assessing against the inclusion and exclusion criteria. Finally, 25 articles were included in this review (Fig 1).

### Study characteristics

Of the 25 studies, 22 were quantitative [25,38–58] and 3 were qualitative [24,59,60]. The studies included in the present review were published between 2010 and 2022. One of these studies was conducted in multiple LDCs, including Chad, Mali, Congo (Brazzaville), Comoros, Laos, Zimbabwe, Burkina Faso, Mauritania, Myanmar, Ghana, Malawi, Ethiopia, and Bangladesh (n = 1) [39], while the rest were conducted in single LDCs, namely Nepal (n = 4) [24,38,54,60], Ethiopia (n = 11) [25,40–42,44,45,48,52,55,57,58], Bangladesh (n = 1) [47], Burkina Faso (n = 1) [43], Tanzania (n = 3) [49,50,53], Uganda (n = 2) [46,51], Lao People’s Democratic Republic (n = 1) [56], and Malawi (n = 1) [59]. The sample size in the individual studies ranged from 72 to 10,021 and the women’s ages ranged from 15 to 72 years. The eligible participants in these studies were recruited from the community (n = 19) [25,38–41,45–53,56,58–60], hospitals (n = 5) [43,44,54,55,57], or both settings (n = 1) [24] (see S2 Table).

### Critical appraisal results

The included studies were appraised using the MMAT. Of the 25 studies, 17 met 100% of the quality criteria; six met 80% of the quality criteria, as they did not report the non-response rate; and two met 60% of the quality criteria, as they did not clearly explain the sampling strategy or sample representativeness of the target population and reported inadequate information about the risk of non-response bias (Table 1). Methodologies and study designs, such as the use of appropriate methods to answer the research questions, sampling strategies, and adherence of different study components to the quality criteria of each method, were well described in the majority of the included studies. Based on the present review question, the identified factors associated with utilising cervical cancer screening services among eligible women in LDCs were categorised into various themes and subthemes.

**Table 1:** Results of quality assessment of included studies based on Mixed Method Appraisal Tool (MMAT).

S.N	Quantitative study	Methodological quality criteria					
		Is the sampling strategy relevant to address the research question?	Is the sample representative of the target population?	Are the measurements appropriate?	Is the risk of nonresponse bias low?	Is the statistical analysis appropriate to answer the research question?	Quality rating
1	Islam et al., 2015	1	1	1	1	1	*****
2	Akinyemiju 2012	1	1	1	–	1	****
3	Ndejjo et al., 2016	1	1	1	1	1	*****
4	Compaore et al., 2016	1	0	1	–	1	***
5	Kileo et al., 2015	1	1	1	–	1	****
6	Bayu et al., 2016	1	1	1	1	1	*****
7	Woldetsadik et al., 2020	1	1	1	–	1	****
8	Perng et al., 2013	1	1	1	1	1	*****
9	Acharya et al., 2017	1	1	1	–	1	****
10	Aynalem et al., 2020	1	1	1	1	1	*****
11	Azene et al., 2021	1	1	1	1	1	*****
12	Belay et al., 2020	1	1	1	1	1	*****
13	Gemeda et al., 2020	1	1	1	1	1	*****
14	Isabirye et al. 2020	1	1	1	–	1	****
15	Kasim et al., 2020	1	1	1	1	1	*****
16	Mboineki et al., 2020	1	1	1	1	1	*****
17	Nigussie et al., 2019	1	1	1	1	1	*****
18	Destaw et al., 2021	1	1	1	1	1	*****
19	Thapa et al., 2018	–	0	1	1	1	***
20	Phaiphichit et al., 2022	1	1	1	–	1	****
21	Chali et al., 2020	1	1	1	1	1	*****
22	Endalew et al., 2020	1	1	1	1	1	*****
**Qualitative study**		**Methodological quality criteria**					
		Is the qualitative approach appropriate to answer the research question?	Are the qualitative data collection methods adequate to address the research question?	Are the findings adequately derived from the data?	Is the interpretation of results sufficiently substantiated by data?	Is there coherence between qualitative data sources, collection, analysis and interpretation?	Quality rating
Darj et al., 2019		1	1	1	1	1	*****
Geire et al., 2020		1	1	1	1	1	*****
Fort et al., 2011		1	1	1	1	1	*****

Yes (1), No (0), Can’t tell (-).

**Remarks:** 5***** or 100% quality criteria met, 4**** or 80% quality criteria met, 3*** or 60% quality criteria met, 2** or 40% quality criteria met, 1* or 20% quality criteria met.

### Cervical cancer screening uptake

Seventeen of the 25 included studies reported the uptake rates of cervical cancer screening among eligible women in LDCs [24,25,38,40–42,44–52,54,55]. Of these studies, nine were conducted in Ethiopia and reported cervical cancer screening uptake rates ranging from 4% to 21% [25,40–42,44,45,48,52,55], and two were conducted in Uganda and reported cervical cancer screening uptake rates of 4.8% and 20.6% [46,51]. Similarly, two studies conducted in Nepal reported screening uptake rates of 18.3% and 13.6% [38,54], one conducted in Bangladesh reported an uptake rate of 8.3% [47], and two studies conducted in Tanzania reported screening uptake rates ranging from 8.2% to 21% [49,50]. Lastly, one study reported a cervical cancer screening rate as low as 4.1% among eligible women from low-income countries [39].

### Factors associated with the uptake of cervical cancer screening

#### Socio-demographic factors.

##### Age.

Nine studies reported that older age was significantly associated with women’s increased uptake of cervical cancer screening [25,40–43,45,47,53,55]. In contrast, one of the included studies reported that younger age was associated with increased uptake of cervical cancer screening [49] and two studies did not find a significant association between age and cervical cancer screening uptake among women from LDCs [38,54].

#### Residential areas.

**Fig 2:**
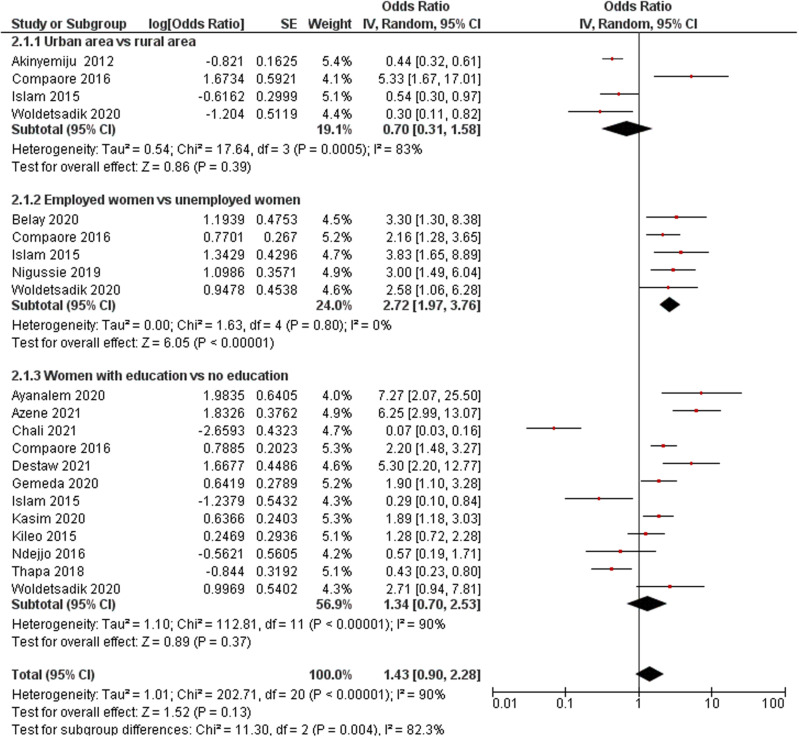
a. Forest plot of women’s residential areas and cervical cancer screening. b. Forest plot of women’s employment status and cervical cancer screening uptake. c. Forest plot of women’s educational status and cervical cancer screening uptake.

All four quantitative studies that reported on the association between women’s residential areas and cervical cancer screening uptake were pooled in the meta-analysis [39,43,47,55]. The findings demonstrated that women living in rural areas were less likely to undergo cervical cancer screening than those living in urban areas (OR: 0.70; 95% CI: 0.31–1.58; *p* = 0.39; *I*^*2*^ = 83%); however, statistical significance was not reached (Fig 2a). Similar findings were observed in qualitative studies indicating that women from rural areas were less likely to have undergone cervical cancer screening than those from urban areas, due to the inadequate availability of healthcare facilities, including screening services for cervical cancer [39,59,60]. In rural areas, poorly equipped examination rooms in healthcare facilities and lack of privacy during examination, e.g., conducting screening on floor mats and examining many women simultaneously, were found to deter women from utilising screening services [58].

#### Financial status.

Six studies examined women’s financial status and uptake of cervical cancer screening [39,43,46,51,53,55]. Four of these six studies reported that women of lower socio-economic status were less likely to undergo cervical cancer screening [39,43,46,55]. In contrast, one study reported that those women who encountered financial barriers were more likely to undergo cervical cancer screening tests than their counterparts [53]. A study by Ndejjo et al. (2016) [51] reported that the financial status of the women had no association with cervical cancer screening uptake.

#### Employment status.

Five studies that investigated women’s employment status were pooled for the meta-analysis [42,43,47,52,55]. The findings demonstrated that employed women were 2.72 times more likely to have undergone screening for cervical cancer than unemployed women (OR: 2.72; 95% CI: 1.97–3.76; *p* < 0.001; *I*^*2*^ = 0%) (Fig 2b).

#### Educational status.

Thirteen studies, namely 12 quantitative studies [40,41,43–45,47–49,51,54,55,58] and one qualitative study [38], examined the association between women’s educational background and cervical cancer screening uptake. All the quantitative studies that reported on this association were pooled in the meta-analysis [40,41,43–45,47–49,51,54,55,58]. The pooled results demonstrated that women with higher educational backgrounds were 1.34 times more likely to have undergone screening than those without such backgrounds (OR: 1.34; 95% CI: 0.70–2.53; *p *= 0.37; *I*^*2*^ = 90%), however, statistical significance was not reached (Fig 2c). In contrast, Acharya et al. [38] reported that women’s educational backgrounds were significantly positively associated with their cervical cancer screening uptake (*p *= 0.01).

#### Knowledge of cervical cancer and its screening.

**Fig 3:**
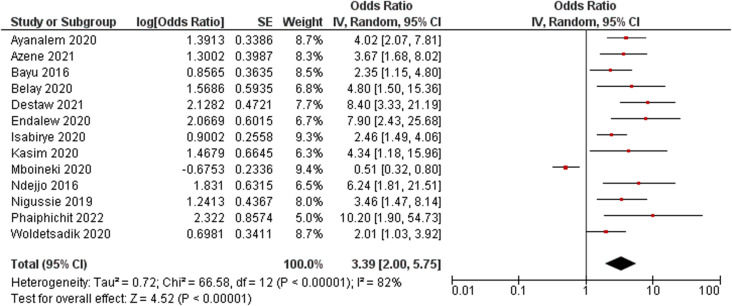
Forest plot of the association between women’s knowledge and cervical cancer screening uptake.

Thirteen studies that investigated women’s knowledge of cervical cancer and its screening were pooled for the meta-analysis of this factor [25,40–42,44,46,48,50–52,55]. The findings showed that women who were knowledgeable about cervical cancer and its screening were more likely to have undergone screening than those with poor or no knowledge (OR: 3.39; 95% CI: 2.00–5.75; *p* < 0.001; *I*^*2*^ = 82%) (Fig 3). Consistent findings were observed in all the qualitative studies included in this review, which reported that a lack of, or inadequate, knowledge of cervical cancer and its screening was directly related to low uptake of cervical cancer screening [24,59,60].

#### Myths and misconceptions about cervical cancer and its screening.

Darj et al. [24] reported several misconceptions about cervical cancer and its screening held by many women in LDCs, which contributed to the reduced uptake of cervical cancer screening: (i) cervical cancer is a deadly and unusual disease with no cure other than surgery and causes substantial stress and emotional and physical suffering to both the patients and their family members; (ii) cervical cancer screening is only used for the detection of cancer that is already present or is usually provided for sick women and those with other uterine complications; (iii) screening should be completed every six months at the nearest health centre; (iv) negative results from preliminary screening suggest that an individual will never develop cervical cancer; (v) cervical cancer can be diagnosed and treated simultaneously during screening; and (vi) cervical cancer might show signs and symptoms even at an early stage. Accordingly, most women believed that if they were not experiencing any symptoms, abnormal discharge, or difficulties during sexual intercourse, they did not need to seek screening services [24].

#### Sexual and reproductive health-related factors.

##### Parity status.

**Fig 4:**
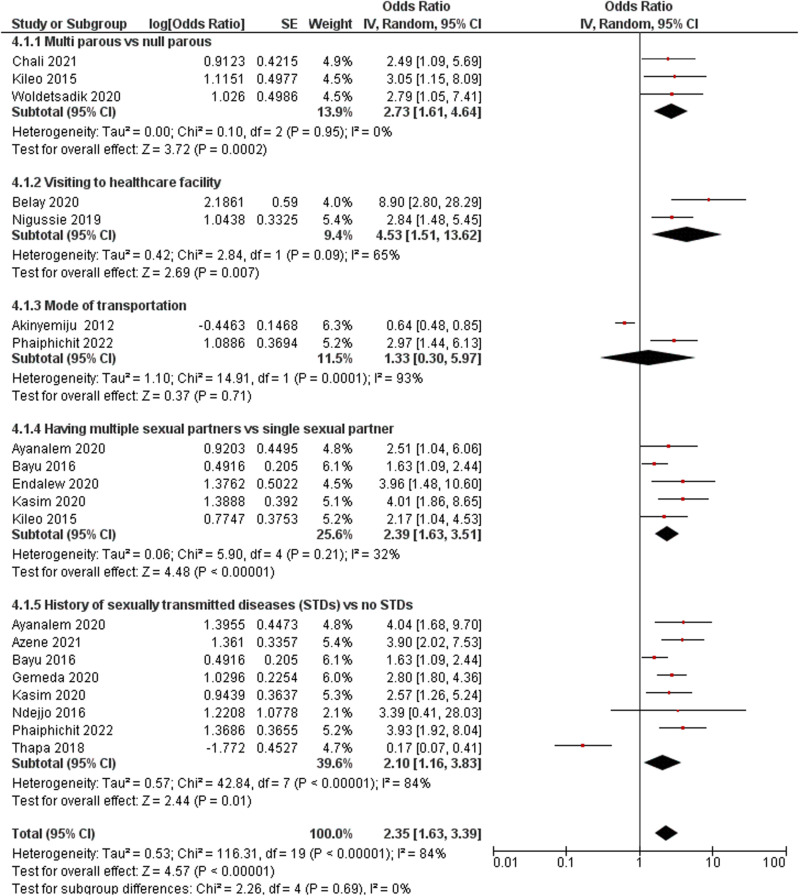
a. Forest plot of the parity status of the women and cervical cancer screening uptake. b. Forest plot of healthcare facility visited and cervical cancer screening uptake c. Forest plot of mode of transportation and cervical cancer screening uptake. d. Forest plot of history of having multiple sexual partners and cervical cancer screening uptake. e. Forest plot of history of having sexually transmitted diseases (STDs) and cervical cancer screening uptake.

Only three studies assessed the parity status of women and cervical cancer screening uptake in women from LDCs [49,55,58]. These three studies were pooled into a meta-analysis, and the pooled results demonstrated that multiparous women were approximately three times more likely to have undergone screening than nulliparous women (OR: 2.73; 95% CI: 1.61–4.64; *p *= 0.002; *I*^*2*^ = 0%) (Fig 4a).

#### Visiting healthcare facilities.

Women who had visited gynaecology units for other examinations were more likely to have undergone screening than those who had never visited gynaecology units (OR: 4.53; 95% CI: 1.51–13.62; *p *= 0.007; *I*^*2*^ = 65%) (Fig 4b) [42,52]. Baley et al. (2020) [42] reported that women who visited private healthcare facilities for reproductive healthcare services and cervical cancer screening uptake were more likely to undergo screening than those who visited public health facilities (OR: 8.9; 95% CI: 2.8–28.9; *p *≤ 0.05). In contrast, women who visited government facilities to receive reproductive healthcare were more likely to undergo cervical cancer screening than women who visited private facilities (OR: 9.71; 95% CI: 1.33–71.11, *p *= 0.025) [51]. Similarly, women who visited non-governmental organisations (NGOs) were less likely to undergo cervical screening tests than those who visited government or private clinics (OR: 0.33, 95% CI: 0.23–1.49; *p *≤ 0.05) [39]. Regarding women’s mode of travel to health facilities, two included studies demonstrated that women who travelled to facilities by car were more likely to be screened for cervical cancer than those who travelled by motorbike or foot (OR: 1.33, 95% CI: 0.30–5.97; *p *= 0.71; *I*^*2*^ = 93%) (Fig 4C) [39,56].

#### History of multiple sexual partners.

Five of the included studies investigated women’s history of multiple sexual partners and uptake of cervical cancer screening, and their results were pooled into a meta-analysis [25,40,48,49,57]. The findings of the meta-analysis demonstrated that women with a history of having multiple sexual partners were 2.39 times more likely to have undergone screening than those without a history of multiple sexual partners (OR: 2.39; 95% CI: 1.63–3.51; *p *< 0.001; *I*^*2*^ = 32%) (Fig 4D).

#### History of sexually transmitted diseases and other illnesses.

Eight studies evaluated the association between previous history of sexually transmitted diseases and uptake of cervical cancer screening [25,40,41,45,48,51,54,56]. The meta-analysis showed that women with a history of sexually transmitted diseases were more likely to have undergone screening than those who had no history of sexually transmitted diseases (OR: 2.10; 95% CI: 1.16–3.83; *p *= 0.01; *I*^*2*^ = 84%) (Fig 4E). This finding is consistent with a qualitative study that demonstrated that women who had chronic abdominal pain were more likely to have undergone screening for cervical cancer than their counterparts [59].

#### Healthcare provider-related factors.

**Fig 5:**
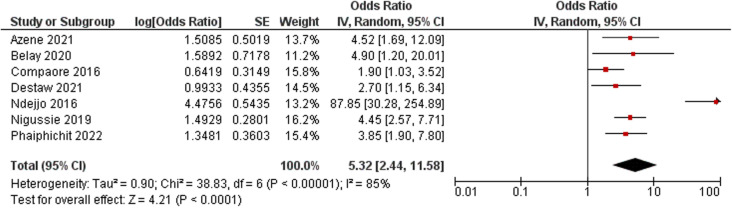
Forest plot of the association between factors related to healthcare providers and cervical cancer screening uptake.

Seven studies that evaluated healthcare provider-related factors and uptake of cervical cancer screening were pooled in a meta-analysis [41–44,51,52,56]. The results showed that women who had received recommendations from healthcare providers were 5.32 times more likely to have undergone cervical cancer screening than those who had never received such recommendations (OR: 5.32; 95% CI: 2.44–11.58; *p *< 0.001; *I*^*2*^ = 85%) (Fig 5). Furthermore, three qualitative studies reported that lack of trust in healthcare providers due to previous uncomfortable screening experiences, inappropriate behaviour by some healthcare providers, and service providers’ negligence and incompetence tended to discourage women from utilising screening services [24,59,60].

#### Decision-making.

The review found that due to the patriarchal nature of society, women in LDCs lack decision-making power, often depending on their husbands and in-laws to decide whether they should access healthcare facilities, including to undergo cervical cancer screening [24,60]. Moreover, women tend to be reluctant to talk about and express their feelings to their husbands and family members because they do not receive permission and encouragement to do so and are afraid of abuse, discrimination, and rejection, which in turn prevents them from seeking screening services [24,60]. In contrast, an included study by Kileo et al. [49] found that women who had not involved their husbands in decision-making regarding healthcare service utilisation were more likely to have undergone cervical cancer screening than those who had involved their husbands in decision-making (OR: 3.73; 95% CI: 2.22–6.26; *p* < 0.005).

#### Perceptions of cervical cancer and its screening.

**Fig 6:**
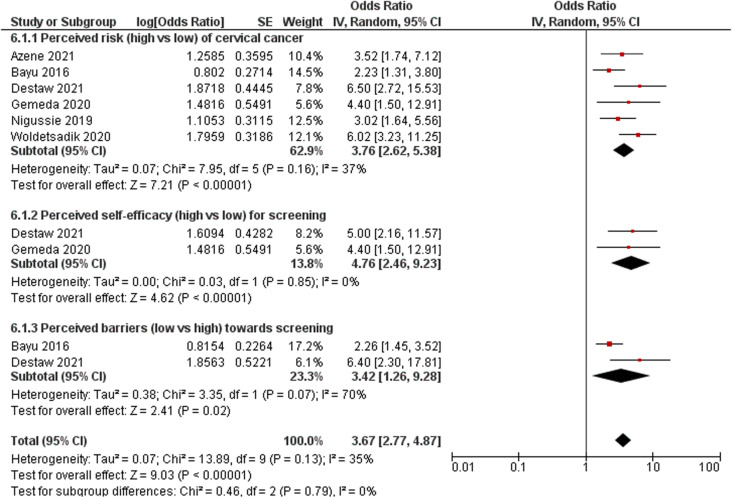
Forest plot of association between women’s perceptions/beliefs and cervical cancer screening uptake.

A total of eight studies reported the association between women’s perceptions of cervical cancer and screening uptake [25,38,41,44,45,52,55,59]. Of these eight studies, six were pooled for meta-analysis, and the results demonstrated that women who perceived a high risk of developing cervical cancer were more likely to undergo screening for cervical cancer test than those who perceived a low risk (OR: 3.76; 95% CI: 2.62–5.38; *p* < 0.001; *I*^*2*^ = 37%) (Fig 6) [25,41,44,45,52,55]. Moreover, women who perceived higher severity and more cues to action were more likely to undergo cervical cancer screening [44,59]. In addition, two studies reported the association between women’s self-efficacy and uptake of cervical cancer screening, and these two studies were pooled for a meta-analysis [44,45]. The finding of the meta-analysis showed that women with high self-efficacy were 4.76 times more likely to have undergone cervical cancer screening than those with low self-efficacy (OR: 4.76; 95% CI: 2.46–9.23; *p* < 0.001; *I*^*2*^ = 0%) (Fig 6). Similarly, the findings also demonstrated that women with lower perceived barriers had higher cervical cancer screening utilisation than their counterparts (OR: 3.42; 95% CI: 1.26–9.28; *p *= 0.02; *I*^*2*^ = 70%) (Fig 6) [25,44]. However, Acharya et al. [38] and Fort et al. [59] reported that cervical cancer screening behaviour was not associated with women’s perceptions of severity, benefits, or barries.

#### Cultural factors.

Various cultural factors, such as fear of discrimination and exclusion from society, influenced women’s decision to undergo cervical cancer screening [24,59,60]. Another factor that prevented women from undergoing screening tests was that male healthcare providers might perform the examinations. Women tended to feel ashamed and embarrassed about showing their genitals to healthcare providers, particularly if they were male [24,59,60]. Moreover, women expressed a sense of discomfort and shame at the idea of other people knowing that they might be ‘sick’ [24]. They feared that people would suspect and assume that they had been cheating on their husbands with multiple sexual partners and were thus ‘being punished for what they had done’ [24]. Women also feared being excluded from social activities such as gatherings for festivals; thus, fear of social rejection was a major barrier to cervical cancer screening utilisation [24,59,60]. These findings suggest that it is imperative to develop and implement culturally appropriate and women-friendly interventions to educate women and their family members regarding the importance of cervical cancer screening and encourage them to participate in such screening to reduce the burden of cervical cancer across the local, community, national, and global levels [24,59,60].

#### Use of mass media.

Women who tended to listen to the radio regularly or at least 1–3 times a week were more likely to have undergone screening than those who never listened to the radio (OR: 24.76; 95% CI: 11.49–53.33; *p* < 0.001) [53].

## Discussion

To the best of our knowledge, this systematic review and meta-analysis is the first to synthesise current evidence regarding cervical cancer screening uptake and its associated factors among women in LDCs worldwide. The findings revealed that the cervical cancer screening uptake rates among eligible women in LDCs were notably lower, ranging from 4% to 21%, than those in developed regions/countries such as Hong Kong (48%) [61] and England (85.7%) [62]. Screening coverage in LDCs is significantly lower than the WHO’s target screening rate of 70% among women aged 35–45 years by 2030 [13]. Our systematic review and meta-analysis further identified multiple factors associated with the low cervical cancer screening uptake rate among eligible women in LDCs, relating to socio-demographic factors, knowledge of cervical cancer and its screening, sexual and reproductive health-related factors, healthcare providers’ recommendations, decision-making power, perceived beliefs and misconceptions regarding cervical cancer and its screening, cultural factors, and use of mass media. Among these factors, knowledge and awareness of cervical cancer and screening tests were positively and significantly associated with cervical cancer screening uptake among women in LDCs. Previous reviews have similarly reported that women with good knowledge of cervical cancer and its screening are more likely to undergo screening than those with poor or no knowledge [9,27]. Thus, it is critical to plan and implement context-specific and culturally appropriate educational programmes targeting women and their family members, including their husbands and community members, to increase screening coverage. It is suggested that women be involved in leading cervical cancer programmes and that these programmes be integrated into various national, community, and school programmes to reach the WHO 2030 target of 70% cervical cancer screening coverage [9,13,63,64]. Our systematic review and meta-analysis also found that factors such as a lack of nationally recommended screening programmes, inadequate standard screening facilities in rural areas, lack of time to travel, and poverty also prevented women from utilising the screening services [7,27,65,66]. These findings indicate that the availability of nationally recommended screening services with well-functioning healthcare facilities that are accessible and affordable to women from rural areas is crucial for enhancing women’s participation in screening [8]. Our review also found that women’s sexual and reproductive health status was associated with screening uptake. For instance, multiparous women and women with a history of various illnesses, including sexually transmitted diseases, were more likely to undergo screening than their counterparts [9,63]. Moreover, as women with multiple sexual partners were more likely to be infected with various sexually transmitted diseases, such as HPV, these women were at a higher risk of developing cervical cancer than their counterparts. This finding echoes that of a previous review [15]. As such women frequently visit healthcare facilities, the screening uptake rate is higher among them than women with no history of sexually transmitted diseases [9,15]. The review also revealed several myths and misconceptions related to cervical cancer and its screening that affect women’s screening uptake. These stemmed from patriarchal social beliefs, a fatalistic view of cervical cancer, shame about being sick, fear of social exclusion, embarrassment, and fear of abuse and rejection. These findings are supported by previous studies [9,27,49]. The barriers and factors associated with stigma and cultural beliefs that prevent cervical cancer screening utilisation could be addressed by introducing HPV self-sampling [13,19]. HPV self-sampling is a comfortable and reliable screening test for cervical cancer, which is well accepted by women from rural areas [64]. Some women prefer to receive screening using the HPV self-sampling technique because the technique is easy to perform, reduces discomfort and embarrassment, and maintains privacy while giving reliable results [8]. Therefore, promoting and introducing HPV self-sampling in LCDs using community resources, such as community health workers, could improve screening participation, particularly among women from stigmatised backgrounds [8,10,19,67].

Moreover, healthcare providers’ recommendations were found to be an important factor encouraging women’s utilisation of screening services. This is consistent with another review’s finding that advice from healthcare providers is a major facilitator of cervical cancer screening uptake in LDCs [15]. In contrast, a lack of trust in healthcare providers due to discomfort experienced while undergoing screening was negatively associated with screening service utilisation [60]. It is vital to provide continued training and professional development for the healthcare providers involved in cervical cancer screening [19]. Furthermore, the results of our systematic review and meta-analysis revealed that women who had decision-making power regarding the utilisation of healthcare services were more likely to utilise screening services than those whose family members, particularly their husbands, were involved in making these decisions. This finding is in line with the results of a study by Petersen et al. (2022) [8].

Moreover, this review identified perceived susceptibility, severity, low perceived barriers, cues to action, and self-efficacy as predictors of cervical cancer screening utilisation. Women who perceived higher susceptibility to or severity of cervical cancer, fewer barriers to undergoing screening, cues to action, and higher self-efficacy in accessing screening may have had higher education, more knowledge of the benefits of screening, and a better understanding of the consequences of cervical cancer, which might have encouraged them to participate in screening. A similar finding was reported in a previous review [9]. Lastly, this review revealed that the use of media, such as radio, plays an important role in promoting the utilisation of cervical cancer screening services. Therefore, it is important to utilise social media to promote or disseminate health messages rapidly at a low cost to a large number of people across geographical locations [68].

### Limitations

This present review has several limitations. First, the studies included in the review covered only one-third of the LDCs worldwide, so the review findings may not be generalisable to women from all LDCs and may not reflect all of the characteristics of women from all LDCs. Second, as the cervical cancer screening uptake was self-reported by women in some studies, their screening uptake rates and effect size estimates of associated factors might be subject to self-report bias. In addition, only studies published in English were included in this review, which may limit understanding of some factors influencing cervical cancer screening uptake; and the results of our review might be subject to publication bias, as studies without significant findings are less likely to be published. Lastly, a high degree of heterogeneity was observed in the included studies as a result of variation in the study populations with different cultural and social backgrounds. Future studies should explore the reasons for this variability and address the issue of heterogeneity to generate robust evidence and guide the development of effective interventions to increase the uptake of cervical cancer screening by women from LDCs.

### Recommendations

The current review has some recommendations for improvements at the research, government, policy, and community levels. At the research level, future studies should explore the effectiveness of culturally tailored interventions in LDCs using behavioural science approaches to understand barriers to and identify strategies for increasing the uptake of cervical cancer screening. At the government level, it is essential for governments of LDCs to invest additional resources in the development of evidence-based, comprehensive national cervical cancer elimination strategies, which should involve all relevant stakeholders, including academia, policymakers, and communities, and be implemented at all levels, from national to local, to reduce the burden associated with cervical cancer [69]. At the policy level, policymakers from the LDCs should work closely with national and international organisations, such as the WHO, to call for the immediate enforcement of accessible and well-structured national guidelines for cervical cancer screening and prevention programmes. Screening programmes should be incorporated into various other health-related programmes, such as women’s health programmes, national immunisation programmes, and family planning programmes, to educate the public about the importance of early cancer detection and regular screening. However, implementing national recommendations is not sufficient to reduce the cervical cancer burden [7]. Effective screening, which involves proper diagnosis, follow-up, and management of positive cases, is crucial to prevent cervical cancer [7,19].

The government should also provide education and training for healthcare providers, including community healthcare workers, to build their capacity to promote cervical cancer screening (based on national guidelines). At the community level, it is crucial to conduct various women-friendly awareness campaigns, such as age-specific educational interventions, health camps, and school-based health programmes, to educate community members about the benefits of routine screening for and early treatment of cervical cancer [21,70]. In addition, awareness and educational programmes should be widely promoted through social media platforms, such as Facebook, and news programmes and advertisements on television and radio. It is crucial to educate family members, particularly husbands, because family support and permission to attend cervical cancer screening tests are essential to encourage women in LDCs to undergo screening [19].

## Conclusions

This review found that the uptake of cervical cancer screening among eligible women in LDCs was significantly low. The low utilisation of cervical cancer screening services was found to be associated with various factors, such as lack of knowledge of cervical cancer screening. It is imperative to develop and implement women-friendly and culturally appropriate health interventions to reduce the healthcare burden of cervical cancer among women in LDCs. It is recommended that the governments of all LDCs invest additional resources in policy advancement and development of cervical cancer prevention programmes that are affordable, acceptable, and accessible to improve screening uptake and thereby reduce cervical cancer morbidity and mortality in their female populations.

## Supporting information

S1 Table Keywords and Search Strategy. (DOCX)

S2 Table Summary of the included studies. (DOCX)

S3 Table PRISMA 2020 Checklist. (DOCX)

S4 Table List of full-text excluded articles (titles and reasons). (DOC)

S5 Table All the included studies identified during the literature search (after duplicate studies removed). (DOCX)

## References

[1] Sung H, Ferlay J, Siegel RL, Laversanne M, Soerjomataram I, Jemal A, et al. Global Cancer Statistics 2020: GLOBOCAN Estimates of Incidence and Mortality Worldwide for 36 Cancers in 185 Countries. CA Cancer J Clin. 2021;71(3):209–49. doi: 10.3322/caac.21660 33538338

[2] Mekuria M, Edosa K, Endeshaw APM, Bala E, Ejeta E, Deriba B, et al. Prevalence of Cervical Cancer and Associated Factors Among Women Attended Cervical Cancer Screening Center at Gahandi Memorial Hospital, Ethiopia. La Revue Sage-Femme. 2021;20:1–6. 10.1177/11769351211068431 PMC8725021 34992337

[3] Dykens JA, Smith JS, Demment M, Marshall E, Schuh T, Peters K, et al. Evaluating the implementation of cervical cancer screening programs in low-resource settings globally: a systematized review. Cancer Causes Control. 2020;31(5):417–29. doi: 10.1007/s10552-020-01290-4 32185604 PMC7105425

[4] Arbyn M, Weiderpass E, Bruni L, de Sanjosé S, Saraiya M, Ferlay J, et al. Estimates of incidence and mortality of cervical cancer in 2018: a worldwide analysis. Lancet Glob Health. 2020;8(2):e191–203. doi: 10.1016/S2214-109X(19)30482-6 31812369 PMC7025157

[5] Cubie HA, Campbell C. Cervical cancer screening - The challenges of complete pathways of care in low-income countries: Focus on Malawi. Womens Health (Lond). 2020;16:1745506520914804. doi: 10.1177/1745506520914804 32364058 PMC7225784

[6] Gravitt PE, Silver MI, Hussey HM, Arrossi S, Huchko M, Jeronimo J, et al. Achieving equity in cervical cancer screening in low- and middle-income countries (LMICs): Strengthening health systems using a systems thinking approach. Prev Med. 2021;144:106322. 33678230 10.1016/j.ypmed.2020.106322 PMC12961690

[7] Bruni L, Serrano B, Roura E, Alemany L, Cowan M, Herrero R, et al. Cervical cancer screening programmes and age-specific coverage estimates for 202 countries and territories worldwide: a review and synthetic analysis. Lancet Glob Health. 2022;10(8):e1115–27. doi: 10.1016/S2214-109X(22)00241-8 35839811 PMC9296658

[8] Petersen Z, Jaca A, Ginindza TG, Maseko G, Takatshana S, Ndlovu P, et al. Barriers to uptake of cervical cancer screening services in low-and-middle-income countries: a systematic review. BMC Womens Health. 2022;22(1):486. doi: 10.1186/s12905-022-02043-y 36461001 PMC9716693

[9] Desta M, Getaneh T, Yeserah B, Worku Y, Eshete T, Birhanu MY, et al. Cervical cancer screening utilization and predictors among eligible women in Ethiopia: A systematic review and meta-analysis. PLoS One. 2021;16(11):e0259339. doi: 10.1371/journal.pone.0259339 34735507 PMC8568159

[10] Reza S, Anjum R, Khandoker RZ, Khan SR, Islam MR, Dewan SMR. Public health concern-driven insights and response of low- and middle-income nations to the World health Organization call for cervical cancer risk eradication. Gynecol Oncol Rep. 2024;54:101460. doi: 10.1016/j.gore.2024.101460 39114805 PMC11305207

[11] United Nations. The least developed countries category: 2024 country snapshots. 2024. Available from: chrome-extension://efaidnbmnnnibpcajpcglclefindmkaj/https://www.un.org/development/desa/dpad/wp-content/uploads/sites/45/2024-Snapshots.pdf

[12] Organization WH. WHO recommends DNA testing as a first-choice screening method for cervical cacner prevention. 2021. Available from: https://www.who.int/europe/news/item/11-09-2021-who-recommends-dna-testing-as-a-first-choice-screening-method-for-cervical-cancer-prevention#:~:text=The%20recently%20published%20%E2%80%9CWHO%20guideline%20for%20screening%20and,about%20342%20000%20women%20died%20from%20the%20disease

[13] World Health Organization. Global strategy to accelerate the elimination of cervical cancer as a public health problem. 2024. Available from: chrome-extension://efaidnbmnnnibpcajpcglclefindmkaj/https://iris.who.int/bitstream/handle/10665/336583/9789240014107-eng.pdf?sequence=1

[14] Islam RM, Billah B, Hossain MN, Oldroyd J. Barriers to Cervical Cancer and Breast Cancer Screening Uptake in Low-Income and Middle-Income Countries: A Systematic Review. Asian Pac J Cancer Prev. 2017;18(7):1751–63. doi: 10.22034/APJCP.2017.18.7.1751 28749101 PMC5648375

[15] Ayenew AA, Zewdu BF, Nigussie AA. Uptake of cervical cancer screening service and associated factors among age-eligible women in Ethiopia: systematic review and meta-analysis. Infect Agent Cancer. 2020;15(1):67. doi: 10.1186/s13027-020-00334-3 33292388 PMC7666476

[16] Nwabichie CC, Manaf RA, Ismail SB. Factors Affecting Uptake of Cervical Cancer Screening Among African Women in Klang Valley, Malaysia. Asian Pac J Cancer Prev. 2018;19(3):825–31. doi: 10.22034/APJCP.2018.19.3.825 29582641 PMC5980862

[17] WHO guideline for screening and treatment of cervical pre-cancer lessions for cervical cancer prevention. World Health Organization; 2021. 34314129

[18] Vega-Crespo B, Neira VA, Maldonado-Rengel R, López D, Delgado-López D, Guerra Astudillo G, et al. “Barriers and Advantages of Self-Sampling Tests, for HPV Diagnosis: A Qualitative Field Experience Before Implementation in a Rural Community in Ecuador”. Int J Womens Health. 2024;16:947–60. doi: 10.2147/IJWH.S455118 38827925 PMC11143988

[19] Bogdanova A, Andrawos C, Constantinou C. Cervical cancer, geographical inequalities, prevention and barriers in resource depleted countries. Oncol Lett. 2022;23(4):113. doi: 10.3892/ol.2022.13233 35251344 PMC8850967

[20] Orang’o EO, Wachira J, Asirwa FC, Busakhala N, Naanyu V, Kisuya J, et al. Factors Associated with Uptake of Visual Inspection with Acetic Acid (VIA) for Cervical Cancer Screening in Western Kenya. PLoS One. 2016;11(6):e0157217. doi: 10.1371/journal.pone.0157217 27310005 PMC4911084

[21] Lee H, Mtengezo JT, Kim D, Makin MS, Kang Y, Malata A, et al. Exploring Complicity of Cervical Cancer Screening in Malawi: The Interplay of Behavioral, Cultural, and Societal Influences. Asia Pac J Oncol Nurs. 2019;7(1):18–27. doi: 10.4103/apjon.apjon_48_19 31879680 PMC6927154

[22] Fentie AM, Tadesse TB, Gebretekle GB. Factors affecting cervical cancer screening uptake, visual inspection with acetic acid positivity and its predictors among women attending cervical cancer screening service in Addis Ababa, Ethiopia. BMC Womens Health. 2020;20(1):147. doi: 10.1186/s12905-020-01008-3 32677933 PMC7366887

[23] Brisson M, Kim JJ, Canfell K, Drolet M, Gingras G, Burger EA, et al. Impact of HPV vaccination and cervical screening on cervical cancer elimination: a comparative modelling analysis in 78 low-income and lower-middle-income countries. Lancet. 2020;395(10224):575–90. doi: 10.1016/S0140-6736(20)30068-4 32007141 PMC7043009

[24] Darj E, Chalise P, Shakya S. Barriers and facilitators to cervical cancer screening in Nepal: A qualitative study. Sex Reprod Healthc. 2019;20:20–6. doi: 10.1016/j.srhc.2019.02.001 31084813

[25] Bayu H, Berhe Y, Mulat A, Alemu A. Cervical Cancer Screening Service Uptake and Associated Factors among Age Eligible Women in Mekelle Zone, Northern Ethiopia, 2015: A Community Based Study Using Health Belief Model. PLoS One. 2016;11(3):e0149908. doi: 10.1371/journal.pone.0149908 26963098 PMC4786115

[26] Shrestha AD, Andersen JG, Gyawali B, Shrestha A, Shrestha S, Neupane D, et al. Cervical cancer screening utilization, and associated factors, in Nepal: a systematic review and meta-analysis. Public Health. 2022;210:16–25. doi: 10.1016/j.puhe.2022.06.007 35863158

[27] Devarapalli P, Labani S, Nagarjuna N, Panchal P, Asthana S. Barriers affecting uptake of cervical cancer screening in low and middle income countries: A systematic review. Indian J Cancer. 2018;55(4):318–26. doi: 10.4103/ijc.IJC_253_18 30829264

[28] Moher D, Shamseer L, Clarke M, Ghersi D, Liberati A, Petticrew M, et al . Preferred reporting items for systematic review and meta-analysis protocols (PRISMA-P) 2015 statement. Syst Rev. 4(1):1. 2015. 10.1186/2046-4053-4-1 PMC4320440 25554246

[29] Helbach J, Pieper D, Mathes T, Rombey T, Zeeb H, Allers K, et al. Restrictions and their reporting in systematic reviews of effectiveness: an observational study. BMC Med Res Methodol. 2022;22(1):230. 35987985 10.1186/s12874-022-01710-w PMC9392276

[30] The EndNote Team. EndNote. EndNote 20 ed. Philadelphia (PA): Clarivate Analytics; 2013.

[31] Hong QN, Fàbregues S, Bartlett G, Boardman F, Cargo M, Dagenais P, et al. The Mixed Methods Appraisal Tool (MMAT) version 2018 for information professionals and researchers. EFI. 2018;34(4):285–91. doi: 10.3233/efi-180221

[32] El-Awaisi A, Joseph S, El Hajj MS, Diack L. A comprehensive systematic review of pharmacy perspectives on interprofessional education and collaborative practice. Res Social Adm Pharm. 2018;14(10):863–82. doi: 10.1016/j.sapharm.2017.11.001 29132909

[33] Hong QN, Fàbregues S, Bartlett G, Boardman F, Cargo M, Dagenais P, et al. The Mixed Methods Appraisal Tool (MMAT) version 2018 for information professionals and researchers. EFI. 2018;34(4):285–91. doi: 10.3233/efi-180221

[34] The Cochrane Collaboration. Review Manager Web (RevMan Web). 2020. [1.22.0]. Available from: https://revman.cochrane.org/#/myReviews

[35] Higgins JPT, Altman DG, Gøtzsche PC, Jüni P, Moher D, Oxman AD, et al. The Cochrane Collaboration’s tool for assessing risk of bias in randomised trials. BMJ. 2011;343:d5928. doi: 10.1136/bmj.d5928 22008217 PMC3196245

[36] Patsopoulos NA, Evangelou E, Ioannidis JPA. Sensitivity of between-study heterogeneity in meta-analysis: proposed metrics and empirical evaluation. Int J Epidemiol. 2008;37(5):1148–57. doi: 10.1093/ije/dyn065 18424475 PMC6281381

[37] Herrera DJ, van de Veerdonk W, Berhe NM, Talboom S, van Loo M, Alejos AR, et al. Mixed-Method Systematic Review and Meta-Analysis of Shared Decision-Making Tools for Cancer Screening. Cancers (Basel). 2023;15(15):3867. doi: 10.3390/cancers15153867 37568683 PMC10417450

[38] Acharya R, Karmacharya E. Cervical cancer screening behavior and associated factors among women of Ugrachandi Nala, Kavre, Nepal. Eur J Med Res. 2017;22(1):32. 28927464 10.1186/s40001-017-0274-9 PMC5606016

[39] Akinyemiju TF. Socio-economic and health access determinants of breast and cervical cancer screening in low-income countries: analysis of the World Health Survey. PLoS One. 2012;7(11):e48834. doi: 10.1371/journal.pone.0048834 23155413 PMC3498259

[40] Aynalem BY, Anteneh KT, Enyew MM. Utilization of cervical cancer screening and associated factors among women in Debremarkos town, Amhara region, Northwest Ethiopia: Community based cross-sectional study. PLoS One. 2020;15(4):e0231307. doi: 10.1371/journal.pone.0231307 32255807 PMC7138328

[41] Azene GK. Visual inspection with acetic-acid (VIA) service utilization and associated factors among women in Hawassa city, southern Ethiopia: a community based cross-sectional study. Womens Midlife Health. 2021;7(1):6. doi: 10.1186/s40695-021-00065-4 34301339 PMC8299609

[42] Belay Y, Dheresa M, Sema A, Desalew A, Assefa N. Cervical Cancer Screening Utilization and Associated Factors Among Women Aged 30 to 49 Years in Dire Dawa, Eastern Ethiopia. Cancer Control. 2020;27(1):1073274820958701. doi: 10.1177/1073274820958701 33034204 PMC7791449

[43] Compaore S, Ouedraogo CMR, Koanda S, Haynatzki G, Chamberlain RM, Soliman AS. Barriers to Cervical Cancer Screening in Burkina Faso: Needs for Patient and Professional Education. J Cancer Educ. 2016;31(4):760–6. doi: 10.1007/s13187-015-0898-9 26336956 PMC4779069

[44] Destaw A, Midaksa M, Addissie A, Kantelhardt EJ, Gizaw M. Cervical cancer screening “see and treat approach”: real-life uptake after invitation and associated factors at health facilities in Gondar, Northwest Ethiopia. BMC Cancer. 2021;21(1):1031. doi: 10.1186/s12885-021-08761-0 34530761 PMC8444493

[45] Gemeda EY, Kare BB, Negera DG, Bona LG, Derese BD, Akale NB, et al. Prevalence and Predictor of Cervical Cancer Screening Service Uptake Among Women Aged 25 Years and Above in Sidama Zone, Southern Ethiopia, Using Health Belief Model. Cancer Control. 2020;27(1):1073274820954460. doi: 10.1177/1073274820954460 32951445 PMC7791476

[46] Isabirye A, Mbonye MK, Kwagala B. Predictors of cervical cancer screening uptake in two districts of Central Uganda. PLoS One. 2020;15(12):e0243281. doi: 10.1371/journal.pone.0243281 33270792 PMC7714132

[47] Islam RM, Bell RJ, Billah B, Hossain MB, Davis SR. Lack of Understanding of Cervical Cancer and Screening Is the Leading Barrier to Screening Uptake in Women at Midlife in Bangladesh: Population-Based Cross-Sectional Survey. Oncologist. 2015;20(12):1386–92. doi: 10.1634/theoncologist.2015-0235 26590177 PMC4679089

[48] Kasim J, Kalu A, Kamara B, Alema HB. Cervical Cancer Screening Service Utilization and Associated Factors among Women in the Shabadino District, Southern Ethiopia. J Cancer Epidemiol. 2020;2020:6398394. doi: 10.1155/2020/6398394 32695167 PMC7354647

[49] Kileo NM, Michael D, Neke NM, Moshiro C. Utilization of cervical cancer screening services and its associated factors among primary school teachers in Ilala Municipality, Dar es Salaam, Tanzania. BMC Health Serv Res. 2015;15:552. doi: 10.1186/s12913-015-1206-4 26666242 PMC4678732

[50] Mboineki JF, Wang P, Dhakal K, Getu MA, Millanzi WC, Chen C. Predictors of uptake of cervical cancer screening among women in Urban Tanzania: community-based cross-sectional study. Int J Public Health. 2020;65(9):1593–602. doi: 10.1007/s00038-020-01515-y 33130908

[51] Ndejjo R, Mukama T, Musabyimana A, Musoke D. Uptake of Cervical Cancer Screening and Associated Factors among Women in Rural Uganda: A Cross Sectional Study. PLoS One. 2016;11(2):e0149696. doi: 10.1371/journal.pone.0149696 26894270 PMC4760951

[52] Nigussie T, Admassu B, Nigussie A. Cervical cancer screening service utilization and associated factors among age-eligible women in Jimma town using health belief model, South West Ethiopia. BMC Womens Health. 2019;19(1):127. doi: 10.1186/s12905-019-0826-y 31660938 PMC6819648

[53] Perng P, Perng W, Ngoma T, Kahesa C, Mwaiselage J, Merajver SD, et al. Promoters of and barriers to cervical cancer screening in a rural setting in Tanzania. Int J Gynaecol Obstet. 2013;123(3):221–5. doi: 10.1016/j.ijgo.2013.05.026 24095307 PMC4291064

[54] Thapa N, Maharjan M, Petrini MA, Shah R, Shah S, Maharjan N, et al. Knowledge, attitude, practice and barriers of cervical cancer screening among women living in mid-western rural, Nepal. J Gynecol Oncol. 2018;29(4):e57. doi: 10.3802/jgo.2018.29.e57 29770627 PMC5981108

[55] Woldetsadik AB, Amhare AF, Bitew ST, Pei L, Lei J, Han J. Socio-demographic characteristics and associated factors influencing cervical cancer screening among women attending in St. Paul’s Teaching and Referral Hospital, Ethiopia. BMC Womens Health. 2020;20(1):70. 32252733 10.1186/s12905-020-00927-5 PMC7137499

[56] Phaiphichit J, Paboriboune P, Kunnavong S, Chanthavilay P. Factors associated with cervical cancer screening among women aged 25-60 years in Lao People’s Democratic Republic. PLoS One. 2022;17(4):e0266592. doi: 10.1371/journal.pone.0266592 35390098 PMC8989294

[57] Endalew DA, Moti D, Mohammed N, Redi S, Wassihun Alemu B. Knowledge and practice of cervical cancer screening and associated factors among reproductive age group women in districts of Gurage zone, Southern Ethiopia. A cross-sectional study. PLoS One. 2020;15(9):e0238869. doi: 10.1371/journal.pone.0238869 32946461 PMC7500695

[58] Chali K, Oljira D, Sileshi T, Mekonnen T. Knowledge on cervical cancer, attitude toward its screening, and associated factors among reproductive age women in Metu Town, Ilu Aba Bor, South West Ethiopia, 2018: community-based cross-sectional study. Cancer Rep (Hoboken). 2021;4(5):e1382. doi: 10.1002/cnr2.1382 33934571 PMC8552000

[59] Fort VK, Makin MS, Siegler AJ, Ault K, Rochat R. Barriers to cervical cancer screening in Mulanje, Malawi: a qualitative study. Patient Prefer Adherence. 2011;5:125–31. doi: 10.2147/PPA.S17317 21448296 PMC3063659

[60] Greibe Andersen J, Shrestha AD, Gyawali B, Neupane D, Kallestrup P. Barriers and facilitators to cervical cancer screening uptake among women in Nepal - a qualitative study. Women Health. 2020;60(9):963–74. doi: 10.1080/03630242.2020.1781742 32643576

[61] So WKW, Wong CL, Chow KM, Chen JMT, Lam WWT, Chan CWH, et al. The uptake of cervical cancer screening among South Asians and the general population in Hong Kong: A comparative study. Journal of Cancer Policy. 2017;12:90–6. doi: 10.1016/j.jcpo.2017.03.015

[62] Ogunwale AN, Coleman MA, Sangi-Haghpeykar H, Valverde I, Montealegre J, Jibaja-Weiss M, et al. Assessment of factors impacting cervical cancer screening among low-income women living with HIV-AIDS. AIDS Care. 2016;28(4):491–4. doi: 10.1080/09540121.2015.1100703 26493859

[63] Yimer NB, Mohammed MA, Solomon K, Tadese M, Grutzmacher S, Meikena HK, et al. Cervical cancer screening uptake in Sub-Saharan Africa: a systematic review and meta-analysis. Public Health. 2021;195:105–11. doi: 10.1016/j.puhe.2021.04.014 34082174

[64] Narasimhamurthy M, Kafle SU. Cervical cancer in Nepal: Current screening strategies and challenges. Front Public Health. 2022;10:980899. doi: 10.3389/fpubh.2022.980899 36466479 PMC9713638

[65] Shrestha G, Mulmi R, Phuyal P, Thakur RK, Siwakoti B. Experiences of cervical cancer survivors in Chitwan, Nepal: A qualitative study. PLoS One. 2020;15(11):e0234834. doi: 10.1371/journal.pone.0234834 33151965 PMC7644025

[66] Li C, Liu Y, Xue D, Chan CWH. Effects of nurse-led interventions on early detection of cancer: A systematic review and meta-analysis. Int J Nurs Stud. 2020;110:103684. 32702568 10.1016/j.ijnurstu.2020.103684

[67] Rana T, Chan DNS, Nguyen KT, Choi KC, So WKW. Effectiveness of Community Health Worker-Led Interventions in Enhancing Colorectal Cancer Screening Uptake in Racial and Ethnic Minority Populations: A Systematic Review and Meta-analysis. Cancer Nursing. 2024;47(5). 10.1097/NCC.0000000000001222 36927698

[68] Plackett R, Kaushal A, Kassianos AP, Cross A, Lewins D, Sheringham J, et al. Use of Social Media to Promote Cancer Screening and Early Diagnosis: Scoping Review. J Med Internet Res. 2020;22(11):e21582. doi: 10.2196/21582 33164907 PMC7683249

[69] Parikh PM, Mullapally SK, Hingmire S, Kamal Uddin AFM, Thinn MM, Shahi A, et al. Cervical Cancer in SAARC Countries. South Asian J Cancer. 2023;12(1):1–8. 10.1055/s-0043-1764227 PMC9966176 36851937

[70] Kirubarajan A, Leung S, Li X, Yau M, Sobel M. Barriers and facilitators for cervical cancer screening among adolescents and young people: a systematic review. BMC Womens Health. 2021;21(1):122. 33757512 10.1186/s12905-021-01264-x PMC7989022

