# Supplementary material for: Determinants of cervical cancer screening utilisation among women in the least developed countries: A systematic review and meta-analysis
Source: PLoS One. 2025 Jun 24;20(6):e0321627. doi: 10.1371/journal.pone.0321627 (PMC12186883; doi:10.1371/journal.pone.0321627)
Supplement: S1 Table — (DOCX) [file pone.0321627.s001.docx]

| cervical cancer OR uterine cancer OR cervical carcinoma OR uterine cervix carcinoma OR uterine cervical neoplasms OR cervix cancer OR gynecological cancer OR gynecological carcinoma |
| --- |
| AND |
| screening OR early detection of cancer OR cervical smear OR Pap test OR Pap smear OR visual inspection with acetic acid OR Papanicolaou test |
| AND |
| Associated factor OR factors OR Predictor OR predictors OR Barrier OR barriers OR Challenge OR challenges OR Obstacle OR obstacles OR Constraint OR constraints OR Issue OR issues OR Problem OR problems OR facilitator OR facilitators OR hurdle OR limit OR curtain OR stop OR Impediments |
| AND |
| low-income countries OR underdeveloped countries OR poor countries OR least developed countries OR Afghanistan OR Angola OR Bangladesh OR Benin OR Bhutan OR Burkina Faso OR Burundi OR Cambodia OR Central Africa Republic OR Chad OR Comoros OR Congo OR Djibouti OR Eritrea OR Ethiopia OR Gambia OR Guinea OR Guinea Bissau OR Haiti OR Kiribati OR Laos OR Lesotho OR Liberia OR Madagascar OR Malawi OR Mali OR Mauritania OR Mozambique OR Myanmar OR Nepal OR Niger OR Rwanda OR  Sao Tome and Principe  OR Senegal OR Sierra Leone OR Solomon Islands OR Somalia OR  South Sudan OR Sudan OR Timor Leste OR Togo OR Tuvalu OR Uganda OR Tanzania OR Yemen OR Zambia |

**S1 Table: Keywords and Search Strategy**

**S1 Table. Keywords**

| S.N | Keywords | NO. | Remarks |
| --- | --- | --- | --- |
| 1 | (cervical cancer or uterine cancer or cervical carcinoma or uterine cervix carcinoma or uterine cervical neoplasms or cervix cancer or gynecological cancer or gynocological carcinoma).ab. | 133508 |  |
| 2 | (screening or early detection of cancer or cervical smear or Pap test or Pap smear or visual inspection with acetic acid or Papanicolaou test or HPV DNA testing).ab. | 1299923 |  |
| 3 | (Associated factor or factors or Predictor or predictors or Barrier or barriers or Challenge or Challenges or Obstacle or obstacles or Constraint or Constraints or Issue or issues or Problem or Problems or facilitator or facilitators or hurdle or limit or curtain or stop or Impediments).ab | 10921384 |  |
| 4 | (low income countries or underdeveloped countries or poor countries or depleted countries or least developed countries or Afghanistan or Angola or Bangladesh or Benin or Bhutan or Burkina Faso or Burundi or Cambodia or Central Africa Republic or Chad or Comoros or Congo or Djibouti or Eritrea or Ethiopia or Gambia or Guinea or Guinea Bissau or Haiti or Kiribati or Laos or Lesotho or Liberia or Madagascar or Malawi or Mali or Mauritania or Mozambique or Myanmar or Nepal or Niger or Rwanda or (Sao Tome and Principe) or Senegal or Sierra Leone or Solomon Islands or Somalia or South Sudan or Sudan or Timor Leste or Togo or Tuvalu or Uganda or Tanzania or Yemen or Zambia).ab. | 515833 |  |
| 5 | 1 &2 | 32713 |  |
| 6 | 3&5 | 146657 |  |
| 7 | 4 & 6 | 472 |  |

**S1 Table. Databases (OVID EMBASE & MEDLINE)**

| S.N | Keywords | NO. | Remarks |
| --- | --- | --- | --- |
| 1 | (397 Cochrane Reviews matching Cervical cancer OR Uterine cancer OR Cervical carcinoma OR Uterine cervix carcinoma OR Uterine Cervical Neoplasms OR Cervix Cancer OR Gynecological cancer OR gynecological carcinoma in All Text AND Screening OR Early detection of cancer OR cervical smear OR Pap test OR Pap smear test OR Visual inspection with acetic acid OR Papanicolaou test in All Text AND Factor OR Predictor OR Barrier OR Challenge OR Obstacle OR Constraint OR Issue OR Problem OR Inhibitor OR Facilitator in All Text AND Low-Income Countries OR Underdeveloped countries OR Poor countries OR Least developed countries OR Afghanistan OR Angola OR Bangladesh OR Benin OR Bhutan OR Burkina Faso OR Burundi OR Cambodia OR Central Africa Republic OR Chad OR Comoros OR Democratic Republic of the Congo OR Djibouti OR Eritrea OR Ethiopia OR Gambia OR Guinea OR Guinea Bassau OR Haiti OR Kiribati OR Laos OR Lesotho OR Liberia OR Madagascar OR Malawi OR Mali OR Mauritania OR Mozambique OR Myanmar OR Nepal OR Niger OR Rwanda OR Sao Tome and Principe OR Senegal OR Sierra Leone OR Solomon Islands OR Somalia OR South Sudan OR Sudan OR Timor Leste OR Togo OR Tuvalu OR Uganda OR United Republic of Tanzania OR Tanzania OR Vanuatu OR Yemen OR Zambia in All Text AND Women OR Female OR Woman OR Disadvantage women in All Text - (Word variations have been searched) | 205 |  |

**S1 Table. Database (**Cochrane Library)

| S.N | Keywords | NO. | Remarks |
| --- | --- | --- | --- |
| 1 | (Cervical cancer or Uterine cancer or Cervical carcinoma or Uterine cervix carcinoma or Uterine Cervical Neoplasms or Cervix Cancer or Gynecological cancer or gynecological carcinoma).mp. [mp=title, abstract, heading word, drug trade name, original title, device manufacturer, drug manufacturer, device trade name, keyword, floating subheading word, candidate term word] | 186075 |  |
| 2 | (Screening or Early detection of cancer or cervical smear or Pap test or Pap smear test or Visual inspection with acetic acid or Papanicolaou test).mp. [mp=title, abstract, heading word, drug trade name, original title, device manufacturer, drug manufacturer, device trade name, keyword, floating subheading word, candidate term word] | 1630088 |  |
| 3 | (Factor or Predictor or Barrier or Challenge or Obstacle or Constraint or Issue or Problem or Inhibitor or Facilitator).mp. [mp=title, abstract, heading word, drug trade name, original title, device manufacturer, drug manufacturer, device trade name, keyword, floating subheading word, candidate term word] | 36702497 |  |
| 4 | (((Low-Income Countries or Underdeveloped countries or Poor countries or Least developed countries or Afghanistan or Angola or Bangladesh or Benin or Bhutan or Burkina Faso or Burundi or Cambodia or Central Africa Republic or Chad or Comoros or Democratic Republic of the Congo or Djibouti or Eritrea or Ethiopia or Gambia or Guinea or Guinea Bassau or Haiti or Kiribati or Laos or Lesotho or Liberia or Madagascar or Malawi or Mali or Mauritania or Mozambique or Myanmar or Nepal or Niger or Rwanda or Sao Tome) and Principe) or Senegal or Sierra Leone or Solomon Islands or Somalia or South Sudan or Sudan or Timor Leste or Togo or Tuvalu or Uganda or United Republic of Tanzania or Tanzania or Vanuatu or Yemen or Zambia).mp. [mp=title, abstract, heading word, drug trade name, original title, device manufacturer, drug manufacturer, device trade name, keyword, floating subheading word, candidate term word] | 2364837 |  |
| 5 | 1 &2 | 47357 |  |
| 6 | 3&5 | 439230 |  |
| 7 | 4 & 6 | 135 |  |

**S1 Table: Database (CINAHL Ultimate)**

| S.N | Keywords | NO. | Remarks |
| --- | --- | --- | --- |
| 1 | cervical cancer[Title/Abstract] OR uterine cancer[Title/Abstract] OR cervical carcinoma[Title/Abstract] OR uterine cervix carcinoma[Title/Abstract] OR uterine cervical neoplasms[Title/Abstract] OR cervix cancer[Title/Abstract] OR gynecological cancer[Title/Abstract] OR gynecological carcinoma[Title/Abstract] | 71231 |  |
| 2 | screening[Title/Abstract] OR early detection of cancer[Title/Abstract] OR cervical smear[Title/Abstract] OR Pap test[Title/Abstract] OR Pap smear[Title/Abstract] OR visual inspection with acetic acid[Title/Abstract] OR Papanicolaou test[Title/Abstract] | 647503 |  |
| 3 | Associated factor[Title/Abstract] OR factors[Title/Abstract] OR Predictor[Title/Abstract] OR predictors[Title/Abstract] OR Barrier[Title/Abstract] OR barriers[Title/Abstract] OR Challenge[Title/Abstract] OR challenges[Title/Abstract] OR Obstacle[Title/Abstract] OR obstacles[Title/Abstract] OR Constraint[Title/Abstract] OR constraints[Title/Abstract] OR Issue[Title/Abstract] OR issues[Title/Abstract] OR Problem[Title/Abstract] OR problems[Title/Abstract] OR facilitator[Title/Abstract] OR facilitators[Title/Abstract] OR hurdle[Title/Abstract] OR limit[Title/Abstract] OR curtain[Title/Abstract] OR stop[Title/Abstract] OR Impediments[Title/Abstract] | 5613206 |  |
| 4 | low-income countries[Title/Abstract] OR underdeveloped countries[Title/Abstract] OR poor countries[Title/Abstract] OR least developed countries[Title/Abstract] OR Afghanistan[Title/Abstract] OR Angola[Title/Abstract] OR Bangladesh[Title/Abstract] OR Benin[Title/Abstract] OR Bhutan[Title/Abstract] OR Burkina Faso[Title/Abstract] OR Burundi[Title/Abstract] OR Cambodia[Title/Abstract] OR Central Africa Republic[Title/Abstract] OR Chad[Title/Abstract] OR Comoros[Title/Abstract] OR Congo[Title/Abstract] OR Djibouti[Title/Abstract] OR Eritrea[Title/Abstract] OR Ethiopia[Title/Abstract] OR Gambia[Title/Abstract] OR Guinea[Title/Abstract] OR Guinea Bissau[Title/Abstract] OR Haiti[Title/Abstract] OR Kiribati[Title/Abstract] OR Laos[Title/Abstract] OR Lesotho[Title/Abstract] OR Liberia[Title/Abstract] OR Madagascar[Title/Abstract] OR Malawi[Title/Abstract] OR Mali[Title/Abstract] OR Mauritania[Title/Abstract] OR Mozambique[Title/Abstract] OR Myanmar[Title/Abstract] OR Nepal[Title/Abstract] OR Niger[Title/Abstract] OR Rwanda[Title/Abstract] OR Sao Tome[Title/Abstract] AND Principe[Title/Abstract] OR Senegal[Title/Abstract] OR Sierra Leone[Title/Abstract] OR Solomon Islands[Title/Abstract] OR Somalia[Title/Abstract] OR South Sudan[Title/Abstract] OR Sudan[Title/Abstract] OR Timor Leste[Title/Abstract] OR Togo[Title/Abstract] OR Tuvalu[Title/Abstract] OR Uganda[Title/Abstract] OR Tanzania[Title/Abstract] OR Yemen[Title/Abstract] OR Zambia[Title/Abstract] | 61639 |  |
| 5 | 1 &2 | 16925 |  |
| 6 | 3 & 4 | 22808 |  |
| 7 | 5 &6 | 153 |  |

**S1 Table. Database (PubMed)**
