# Supplementary material for: Determinants of cervical cancer screening utilisation among women in the least developed countries: A systematic review and meta-analysis
Source: PLoS One. 2025 Jun 24;20(6):e0321627. doi: 10.1371/journal.pone.0321627 (PMC12186883; doi:10.1371/journal.pone.0321627)
Supplement: S2 Table — (DOCX) [file pone.0321627.s002.docx]

| **Quantitative study** | | | |
| --- | --- | --- | --- |
| **Study /year/ country** | **Study design** | **Study population**  **&**  **sample size (n)** | **Result** |
| Akinyemiju  2012  Chad, Mali, Congo (Brazzaville), Comoros, Laos, Zimbabwe, Burkina Faso, Nepal, Mauritania, Myanmar, Ghana, Malawi, Ethiopia, and Bangladesh | Cross-sectional study  Community setting | Women aged between 18 and 69 years  N=10021 | Uptake rate of cervical cancer screening = 4.1%Factors associated with utilization of cervical cancer screeningResiding in the lowest socio-economic status (SES) households were associated with a 43% of reduction (OR: 0.57; 95% CI: 0.39-0.82).Residing in the middle socio-economic status (SES) household was associated with a 33% reduction in likelihood of receiving a Pap test (OR: 0.66; 95% CI: 0.48-0.09).Visiting non-governmental organization (NGO) instead of a government or private clinic was associated with reduced likelihood of receiving a pap test (OR: 0.33; 95% CI: 0.23-0.49).Residing in a rural area was associated with reduced likelihood of receiving a pap test (OR: 0.44; 95% CI: 0.32-0.60).Travelling to facilities by walking/ bicycle was associated with reduced likelihood of receiving a pap test (OR: 0.64; 95% CI: 0.48-0.86).Travel time was not associated with Pap test uptake. |
| Woldetsadik et al., 2020  Ethiopia | Cross-sectional study  Hospital setting | Women aged between 18 and 47 years  N=425 | Uptake rate of cervical cancer screening = 12.2%Factors associated with utilization of cervical cancer screening **Sociodemographic factors:**   - Women who lived in rural area were less likely to be screened than women who live in urban area (OR: 0.30; 95% CI: 0.11-0.85). - Women aged 40–49 years were more likely to undergo cervical cancer screening practice compared with women aged 18–29 years old (OR: 3.58; 95% CI: 1.21-10.58). - Self-employed women were more likely to be screened than governmental employed women (OR: 2.58; 95% CI: 1.06-6.27). - Compared with nulliparous women, multiparous women were more likely to undergo cervical cancer screening (OR: 2.79; 95% CI: 1.05-7.39). - Women who had earned 3000–3999 Birr (111-148 USA dollar) per month (OR: 2.93; 95% CI: 1.32-6.51). - Women who had earned greater than 4000 Birr (148 USA dollar) were more likely seeking for screening than women who had been earned less than 2000 Birr (75 USA dollar) per month (OR: 2.97; 95% CI: 1.27-6.92). - Marital status and educational level of respondents did not show significant association (P > 0.05).   **Knowledge and perception related factors:**   - Women who heard about cervical cancer screening benefits were more likely to be screened than those women who had not (OR: 4.11; 95% CI: 1.12-15.04). - Women who knew about the precancerous cervical dysplasia which could happen without symptoms were more likely to be screened than women who didn’t know (OR: 2.09; 95% CI: 1.06-4.10). - Women who agreed by the thought that “I can’t do screening even though beneficial” were less likely to be screened as compared with counterparts (OR: 0.10; 95% CI: 0.02-0.43). - Women who likely felt the possibility of getting cervical cancer were more likely to be screened than their counterparts (OR:5.36; 95% CI: 2.12-13.55). - Women who perceived the chance of getting cervical cancer were more likely to be screened as compared with women who unlikely perceived (OR: 3.40; 95% CI: 1.51-7.69) - Women who fear the test outcome of cervical cancer were less likely to be screened than women who did not fear (OR: 0.47; 95% CI: 0.23-0.92). - Women who had more information about cervical cancer were more likely to be screened as compared with women who need more information (OR: 2.01; 95% CI: 1.03-3.95). - Women who knew the place of screening services were more likely to be screened than those who did not know (OR: 11.83; 95% CI: 3.54-39.51). - Women who had a history of cervical illness were more likely to be screened as compared with women who felt healthiness (OR: 8.64; 95% CI: 2.36-31.63). |
| Bayu et al., 2016  Ethiopia | Cross-sectional study  Community setting | Women aged 21 or above  N=1286 | Uptake rate of cervical cancer screening = 19.8%Factors associated with utilization of cervical cancer screening  - Women aged 30-39 years about 1.79 times more likely to be screened for cervical cancer compared with those aged 21-29 years (AOR: 1.79; 95% CI: 1.18-2.73). - Women having history of multiple sexual partners were more likely to get screened when compared to their counterparts (AOR: 1.63; 95% CI: 1.09-2.44). - Women with history of sexually transmitted disease were more likely to get screened for cervical cancer when compared to those who have not experienced the disease (AOR: 4.12; 95% CI: 2.28-7.47). - Women tested positive for HIV were 5.61 times more likely to be screened when compared with those who had tested negative (AOR: 5.61; 95% CI: 2.59-12.14). - Women who perceived higher susceptible to develop cervical cancer were more likely to undergo screening when compared to their counterparts (AOR: 2.22; 95% CI: 1.30-3.78). - Women having low perceived barriers were more likely to be screened than those who have high perceived barriers (AOR: 2.25; 95% CI: 1.44-3.51). - Women who had better knowledge about cervical cancer and its screening were more likely to get screened when compared to those women with no knowledge (AOR: 2.35; 95% CI: 1.15-4.80). |
| Compaore et al., 2016  Burkina Faso | Cross-sectional study  Hospital setting | Women aged between 18 and 72 years  N=351 | Factors associated with utilization of cervical cancer screening  - Higher educated women were more likely to be screened than the women with low and medium level of education (OR: 2.20; 95 % CI: 1.48-3.23). - Older women were more likely to get screened than the women of younger age (OR: 1.10; 95 % CI: 1.06-1.12). - Women from higher socioeconomic status were (SES) more likely to be screened than the women from lower socioeconomic status (OR: 1.50; 95 % CI: 1-2.37). - Employed women were two times more likely to get screening when compared to their counterparts (OR: 1.90; 95 % CI: 1.13-3.11). - Women residing in urban areas were more likely to be screened when compared to women who live than semirural and rural areas (OR: 2.00; 95 % CI :1.19-3.25). - Women who received recommendation/ advice from healthcare providers were more likely to be screened than their counterparts (OR: 1.98; 95 % CI: 1.06-3.69). |
| Perng et al., 2013  Tanzania | Cross-sectional study  Community setting | Women aged 25 or above    N=300 | Factors associated with utilization of cervical cancer screening  - Older women of aged 40–49 years were more likely to be screened for the cervical cancer than the women aged 20–29 years (OR: 4.29; 95% CI: 1.61-11.48; p < 0.02). - Women who listened radio 1-3 times per week were more likely to be screened when compared to women who never listened radio (OR: 24.76; 95% CI: 11.49-53.33; p < 0.001). - Women having poor quality of life were less likely to get screening when compared to women with higher quality of life (OR: 4.91; 95% CI: 1.96-12.32; p <0.001). - Women who faced cost barriers were more likely to get screening when compared to their counterparts (OR: 2.24; 95% CI: 1.11-4.53; p < 0.001). - Women having more positive attitude toward cervical cancer screening were more likely to undergo screening for cervical cancer when compared to their counterparts (OR: 4.64; 95% CI: 1.39-15.55; p <001). |
| Thapa et al., 2018  Nepal | Cross-sectional study  Hospital setting | Women aged 20 or above  N=360 | Uptake rate of cervical cancer screening = 13.6%Factors associated with utilization of cervical cancer screening  - Women’s age was not significantly associated with cervical cancer screening (OR: 0.89; 95% CI: 0.46-1.71; *p*=0.728). - Women’s marital status was not associated with screening uptake of cervical cancer (OR: 1.80; 95% CI: 0.61-5.27; *p*=0.274). - Education status of the women was also not associated with screening uptake (OR: 0.43; 95% CI: 0.23-0.80; *p*=0.007). - Ethnicity of the women was associated with the screening uptake; women belong to Brahmin and Chhetri ethnicity were less likely to be screened than the other ethnicity (OR: 4.68; 95% CI: 2.49-8.80; p <0.001). - Having family history of cancer was associated with screening uptake, women who had family history of having cancer were more likely to be screened than their counterparts (OR: 0.17; 95% CI: 0.07-0.39; *p<*0.001). |
| Kileo et al., 2015  Tanzania | Cross-sectional study  Community Setting | Women aged 20 or above  N=512 | Uptake rate of cervical cancer screening = 21%Factors associated with utilization of cervical cancer screening  - Multiparous women were 3 times more likely to utilize the service compared to those with null parous women (AOR: 3.05; 95 % CI: 1.15-8.06; *p<*0.025). - Women having more lifetime sexual partners were found to be 2 times more likely to utilize cervical cancer screening services compared to those with one lifetime sexual partner (AOR: 2.17; 95 % CI: 1.04-4.54; *p<*0.038). - Women who were aged 40–49 years less likely to utilize the screening services compared to those women aged 20–29 years (AOR: 0.51; 95 % CI: 0.26–0.99; *p<*0.047). - Education level and use of contraceptives were not associated with self-report utilization of cervical cancer screening service - Marital status is not associated with the cervical cancer screening - The negative experience towards the cervical cancer screening reported by some women was not associated with the uptake of the service. - Women who made decision and did not involve their spouse in making the decision to utilize healthcare facilities were 4 times more likely to screen for cervical cancer compared to those who involved their spouse in decision-making (AOR: 3.73; 95 % CI: 2.22-6.26; *p<*0.001). |
| Nigussie et al., 2019  Southwest Ethiopia | Cross-sectional  Community setting | Women aged between 30 and 49 years  N=737 | Cervical cancer screening uptake rate= 15.5%Factors associated with utilization of cervical cancer screening  - Government employed women were more likely to be screened when compared to housewife (AOR: 3.00; 95% CI: 1.49-6.01). - Women who knew someone had undergone screening were more likely to be screened when compared to their counterparts (AOR: 3.61; 95% CI: 2.07-6.29). - Women with history of gynecological examination were more likely to be screened than those without the history of gynecological examination (AOR: 2.84; 95% CI: 1.48-5.45). - Women who did not prefer physician’s gender for gynecological examination where more likely to be screened when compared to the women who preferred physician gender (AOR: 3.57; 95% CI: 1.98-6.45). - Women who received advice from health care providers were more likely to be screened when compared to women who did not receive any recommendation from the healthcare providers (AOR: 4.45; 95% CI: 2.57-7.70). - Women with higher knowledge of cervical cancer screening was positively associated with increased uptake of cervical cancer screening (AOR: 3.47; 95% CI: 1.47-8.21). - Women having high perceived susceptible for cervical cancer was associated with higher screening uptake (AOR: 3.03; 95% CI: 1.64-5.56). |
| Kasim et al., 2020  Ethiopia | Cross-sectional study  Community setting | Women aged between 30 and 49 years  N=506 | Uptake rate of cervical cancer screening = 10.3%Factors associated with utilization of cervical cancer screening  - Women with higher education were about 2 times more likely to undergo cervical cancer screening than those women who never attended any formal education (AOR: 1:89; 95% CI: 1:18-3.05; *p* < 0:009). - Women having history of sexually transmitted disease were about 2.6 times more likely to utilize cervical cancer screening when compared to their counterparts (AOR 2:57; 95% CI: 1:26-5.23; *p* <0.021). - Women having history of multiple sexual partners were 4 times more likely to undergo cervical cancer screening when compared to women with a single partner (AOR: 4:01; 95% CI:1.86-8.66; *p* <0.001). - Women who knew that cervical cancer is a preventable disease were 4.3 times more likely to utilize cervical cancer screening service than those who do not know that cervical cancer is a preventable disease (AOR: 4:34; 95% CI: 1:18-13.05; *p* <0.001). |
| Mboineki  et al., 2020  Tanzania | Cross-sectional study  Community setting | Women aged 21 years or above  N=1013 | Uptake rate of cervical cancer screening = 8.2%Factors associated with utilization of cervical cancer screening  - Women with no screening intention were less likely to be screened than the women with good intention for screening test for cervical cancer (AOR: 0.48; 95% CI: 0.305- 0.761; *p* < 0.002). - Women with poor knowledge were less likely to be screened than women with good knowledge about cervical cancer screening (OR: 0.50; 95% CI: 0.322- 0.806; *p* < 0.004). - Women with positive health beliefs were more likely to uptake cervical cancer screening compared to respondents with negative health beliefs (OR: 0.16; 95% CI: 0.091- 0.293; *p* < 0.001). |
| Belay et al., 2020  Eastern Ethiopia | Cross- sectional study  Community setting | Women aged between 30 and 49 years  N=601 | Cervical cancer screening uptake rate= 4.0Factors associated with utilization of cervical cancer screening  - Women aged 40-49 years were almost four times more likely to use the screening service compared with women aged 30-39 years (AOR: 4.20; 95% CI: 1.3-13.8). - Women who attended the private health facilities were approximately nine times more likely to use screening services compared with those who attended public health facilities (AOR: 8.90; 95% CI: 2.8- 28.0). - Government employed women were three times more likely to utilize the service compared with others (AOR: 3.30; 95% CI: 1.3- 8.8). - The women who visited the gynaecology unit were almost four times more likely to undergo cervical cancer screening (AOR: 3.80; 95% CI: 1.5- 9.8). - Women with good knowledge about cervical cancer and its screening were five times more likely to undergo screening than their counterparts (AOR: 4.80; 95% CI: 1.5- 15.5).. - Women who received advice from healthcare providers were four times more likely to utilize cervical cancer screening (AOR: 4.10; 95% CI: 1.5-11.3). |
| Aynalem et al., 2020  North Ethiopia | Cross-sectional study  Community Setting | Women aged between 30 and 49 years  N=822 | Cervical cancer screening uptake rate= 5.4%Factors associated with utilization of cervical cancer screening  - Women aged 40–49 years were 3.12 times more likely to utilize cervical cancer screening as compared to women aged of 30–39 years (AOR: 3.12; 95% CI: 1.24- 7.84). - Single/divorced/widowed women 3.41 times more likely to be screened for cervical cancer as compared with married women (AOR: 3.41; 95% CI: 1.29-8.97). - Educated women 7.26 times more likely utilized as compared with women who never been to school (AOR: 7.27; 95% CI: 2.07-25.51). - Women who attended secondary education 4.57 times more likely utilized as compared with women who did not attend formal education (AOR: 4.57; 95% CI: 1.19-17.6). - Engaged in sexual activities at an early age before 16 years were 3.02 times more likely to be screened for cervical cancer screening as compared to their counterparts (AOR: 3.02; 95% CI; 1.84- 4.97). - Women having history of multiple sexual partners were 2.51 times more likely to utilize cervical cancer screening as compared to who did not have (AOR: 2.51; 95% CI: 1.04-6.06). - Women having history of STDs were 4.03 times more likely to participate in cervical cancer screening services as compared to those who did not have STDs history (AOR: 4.03; 95% Cl: 1.68-9.72). - Women with good knowledge about cervical cancer screening were 4.02 times more likely to utilize cervical cancer screening service as compared to those who were not knowledgeable (AOR: 4.02; 95% CI: 2.07-7.77). - Women having favourable attitude towards cervical cancer screening were 3.22 times more likely to utilize cervical cancer screening service as compared to those who had unfavourable attitudes (AOR: 3.22; 95% CI: 2.52- 4.12). |
| Gemeda et al., 2020  Ethiopia | Cross sectional study  Community setting | Women aged 25 years or above  N=838 | Cervical cancer screening uptake rate= 17.8%Factors associated with utilization of cervical cancer screening  - Women aged 35-39 years were 5 times more likely have cervical cancer screening uptakes when compared to women with women aged 25-29 years (AOR: 5.20; 95% CI: 2.6-10.6). - Educated women were more likely undergo cervical cancer screening than who have attended no formal education (AOR: 1.90; 95% CI: 1.10-3.20). - Women tested for HIV were nearly 3 times more likely undergo cervical cancer screening when compared to their counterparts (AOR: 2.80; 95% CI: 1.8-4.4). - Respondents who perceived high self-efficacy were 4 times more likely to undergo cervical cancer screening uptakes when compared with perceived low self-efficacy (AOR: 4.40; 95% CI: 1.5-12.8). |
| Islam et al. 2015  Bangladesh | Cross sectional study  Community setting | Women aged between 30 and 59 years  N= 1590 | Cervical cancer screening uptake rate= 8.3%Factors associated with utilization of cervical cancer screening  - Being employed outside the home was strongly associated with screening (AOR: 3.38; 95% CI: 1.65-8.92; p< 0.002). - Women aged 40-49 years were more likely to have been screened than women aged 30-39 years (AOR: 2.17; 95% CI: 1.19-3.94; p<0.011). - Women were less likely to have been screened if they lived in a rural area (AOR: 0.54; 95% CI: 0.30-0.98; p<0.04). - Women having no education was negative associated with the screening uptake (AOR: 0.29; 95% CI: 0.10-0.85; p<0.02). - The marital status, religion, husband’s occupation and body mass index were not associated with screening uptake |
| Ndejjo et al. 2016  Uganda | Cross-sectional study  Community setting | Women aged between 25 and 49 years  N= 900 | Cervical cancer screening uptake rate= 4.8%Factors associated with utilization of cervical cancer screening  - Women who received recommended from health providers were 87 times more likely to undergo screening for cervical cancer than their counterparts (AOR: 87.85; 95% CI: 30.28-254.840; p< 0.001). - Women who knew about screening providers were 6 times more likely to have undergone the procedure (AOR: 6.24; 95% CI: 1.81-21.56; p< 0.004). - Women who knew someone had ever been screened were 9 times more likely to have screened for cervical cancer (AOR: 9.48; 95% CI: 2.39-37.56; p< 0.001). - Women’s residency areas such as rural, semi urban and urban was not significantly associated with cervical cancer screening uptake. - Women age, education level, occupation, income, HIV testing status and number of people in household were not significantly associated with cervical cancer screening uptake. - The facility where women accessed reproductive health care, knew that cervical cancer can be prevented, knew more than one preventive measure for cervical cancer, knew at least one test for cervical cancer, and knew someone who had ever been diagnosed with cervical cancer were not significantly associated with cervical cancer screening uptake. |
| Isabirye et al., 2020    Uganda | Cross-sectional study  Community Setting | Women aged 25-49 years,  N=845 | Cervical cancer screening uptake rate= 20.6 %Factors associated with utilization of cervical cancer screening  - Rich women were more likely to be screened for cervical cancer compared to the poor women (AOR: 1.90; 95% CI: 1.06-3.42; p< = 0.031). - Women who received information from healthcare provider were more likely to undergo screening when compared to those women whose main source of information were radios (AOR: 4.14; 95% CI: 2.65-6.48; p< 0.001). - Women with good knowledge about cervical cancer and its screening were more likely to get screening for cervical cancer when compared to their counterparts (AOR: 2.25; 95% CI: 1.49-3.37; p< 0.001). |
| Acharya et al., 2017  Nepal | Cross sectional study  Community setting | Women aged 30 to 60 years,    N=180 | Cervical cancer screening uptake rate= 18.3%Factors associated with utilization of cervical cancer screening  - Women’s age (*p* = 0.698) and parity (*p* = 0.142) had no association with cervical cancer screening behaviour. - Women’s education (*p* = 0.015) had no significant association with cervical cancer screening behaviour. - Perceived severity towards cervical cancer and its screening (*p* = 0.245) has no association with cervical cancer screening behaviour. - Perceived benefits towards cervical cancer screening (*p*=0.075) had no association between cervical cancer screening behaviour. - Perceived barriers (*p* = 0.310) towards cervical cancer and its screening had no association with cervical cancer screening behavior. |
| Destaw et al., 2021  Ethiopia | Cross-sectional  Study  Hospital setting | Women aged between 30-49 years  N=464 | Cervical cancer screening uptake rate= 16.4%Factors associated with utilization of cervical cancer screening  - Educated women were more likely to undergo cervical cancer screening than women without education (AOR: 5.30; 95% CI: 2.20-13.0). - Women who perceived a high susceptibility to cervical cancer were more likely to undergo screening than women who had low perceived susceptibility (AOR: 6.50; 95% CI: 2.72-15.51; p<0.001). - Women who had low perceived barriers were more likely to undergo cervical cancer screening than women with high perceived barriers (AOR: 6.40; 95% CI: 2.30-17.80; p<0.001). - Women who high perceived severity of cervical cancer were more likely to undergo screening than those who had low perceived severity (AOR: 3.40; 95% CI: 1.01- 11.65; p<0.04). - Women who had high cues to action were more likely to undergo cervical cancer screening than women who had low cues to action (AOR: 4.50; 95% CI: 1.86-11.32). - Women with high self-efficacy were more likely to undergo screening test for cervical cancer than those with low self-efficacy (AOR: 5.00; 95% CI: 2.16-11.6; p<0.001). - Women with good knowledge regarding cervical cancer and screening were more likely to undergo the screening test (AOR: 8.40; 95% CI: 3.33-21.21; p<0.001). - Finally, women who received recommendation by healthcare providers before were more likely to undergo cervical cancer screening (AOR: 2.70; 95% CI: 1.15- 6.51; p<0.02). |
| Azene  2021  Southern Ethiopia | Cross-sectional study  Community setting | Women aged between 30-49 years  N= 411 | Cervical cancer screening uptake rate= 20.7%Factors associated with utilization of cervical cancer screening  - Women aged 40-49 years were almost 5 times likely to be screened for cervical cancer when compared to women aged 30-39 years (AOR: 4.64; 95% CI: 2.15-10.01; p<0.001). - Women with history of having sexually transmitted diseases (STDs) were almost 4 times likely to undergo cervical cancer screening when compared with the women without history of STDs (AOR: 3.90; 95% CI: 2.02-7.53; p<0.001). - Women who received recommendation from healthcare providers were almost 4.5 times more likely to get screening for cervical cancer when compared to their counterparts (AOR: 4.52; 95% CI: 1.70-12.11; p<0.001). - Women with good knowledge and awareness about the cervical cancer and its screening were 4 times more likely to be screened for cervical cancer when compared to women with no knowledge and awareness (AOR: 3.67; 95% CI: 1.68-8.04; p<0.001). - Women with high perceived self-risk about cervical cancer were 3.5 times more likely to undergo for cervical cancer when compared to their counterparts (AOR: 3.52; 95% CI: 1.74-7.13; p<0.001). - Women who received community health education were more likely to get screened when compared to those women without community health education (AOR: 6.25; 95% CI: 2.10-13.05; p<0.001). |
| Phaiphichit et al. 2022  Lao People’s Democratic Republic | Case-control study  Community setting | Women  aged 25-60 years  N=36 | Women with history of having symptoms of STIs had higher cervical cancer screening uptake than women without history of STIs (AOR: 3.93; 95% CI: 1.92-8.05; p<0.001).Women who received recommendation from health worker had increased uptake of cervical cancer screening when compared to women who did not receive recommendation from health worker (AOR: 3.85; 95% CI: 1.90-7.78; p<0.001).Women who had higher level of knowledge about cervical cancer had higher cervical cancer screening uptake compared to those who had lower level of knowledge (AOR: 7.90; 95% CI: 2.43-25.69; p=0.001).Women who had good attitude toward cervical cancer had greater uptake of cervical cancer when compared to those who had poor attitude towards cervical cancer (AOR: 7.90; 95% CI: 1.48-4.30; p=0.013).Women who travelled by car to the health facility had higher uptake of cervical cancer screening when compared to women who travelled by motorbike to health facility (AOR: 2.97; 95% CI: 1.44-6.11; p=0.003). |
| Endalew  et al. 2020  Ethiopia | Cross-sectional study  Hospital setting | Reproductive age group  (15-49 years)  N=260 | Women who had experience of having sex aged between 15-17 years were more likely to undergo cervical cancer screening when compared to those who had first sexual experience in other age (AOR: 6.05; 95% CI: 1.167-31.36).  - Women who had the information about cervical cancer had higher uptake of cervical cancer screening compared to those without information about cervical cancer (AOR: 10.20; 95% CI: 1.90-96.40). - Similarly, women who had more than one sexual partner were more likely to be screened with cervical cancer when compared to those women without multiple sexual partners (AOR: 3.96; 95% CI: 1.48-10.58). |
| Chali et al. 2021  Ethiopia | Cross sectional study  Community setting | Reproductive age group women  N=420 | Women who can’t read and write were 97% times less likely to have positive attitude toward cervical cancer screening when compared to those with college and above educational level (AOR: 0.03; 95% CI: 0.01-0.10).  - Women who were able to read and write were 95% times less likely to have positive attitude toward cervical cancer screening when compared with college and above educational level (AOR:0.05; 95% CI:0.02-0.15). - Women with primary education were 93% times less likely to have a positive attitude toward cervical cancer screening when compared to women with college and above educational status (AOR: 0.07; 95% CI: 0.03-0.17). - Similarly, multi-parous women were more likely to have a positive attitude toward cervical cancer compared to null-parous women (AOR: 2.49; 95% CI: 1.09-5.72). |

| **Qualitative study** | | | |
| --- | --- | --- | --- |
| **First author/ year** | **Study Design** | **Study participants**  **/Sample size** | **Results** |
| Greibe et al., 2020  Nepal | Qualitative study  (Focus group discussion and in-depth interview)  -  Community setting | Women aged between 30 and 60 years  N=48 | **Husband’s support for screening**   - Women lacked support and encouragement from husbands to get screening for cervical cancer. - Women had to depend on her husband and family to make decision to get screened for cervical cancer. - Husbands lack of knowledge of risk factors and benefits was found to be associated with lack of support and encouragement for screening. - Women was afraid of being rejected and abuse from husband and society if they detected with cervical cancer because screening might detect a cancer which result in social exclusion.     **Stigma and discrimination**   - Women was afraid of being discriminated by husbands and family because the family and husband thought she would be a burden for them if she diagnosed with cervical cancer. - Fear of social exclusion and gossip such as “members of community say that the women is a selfish devil, and that’s why she got ill and” being punished for what she has done” were found be the barriers for women to undergo screening for cervical cancer. - Misconceptions and limited knowledge were found to be associated with poor uptake of cervical cancer screening. - Reproduction issues was found to be associated with screening uptake because the family and community would think if women diagnosed with cervical cancer, then she would never be able to give birth.   **Awareness about screening options**   - Poor knowledge about the cervical cancer and its screening was found to be associated with low uptake of cervical cancer screening. - Only seeking health care when having symptoms “if there is no pain then it is not necessary to go for screening”   **Logistics and Financial Issues**   - Travelling cost for transportation and treatment cost was related to poor screening uptake. - Women with positive experience with screening were more likely to be screened than those with negative screening experience. - Women with bad experience of screening in which screening was performed on mats on the floor without any beds. This would discourage women to undergo screening for cervical cancer. - Unstandardised screening procedures were negatively associated with screening uptake among the women.   **Health care providers**   - Lack of trust was negatively associated with screening uptake “If confidentiality is not maintained the probability of women getting screened reduce”. - Being examined by female doctor or nurse was positively associated with high screening uptake. Women feel ashamed to be examined by the male healthcare providers. |
| Fort et al., 2011  Malawi | Qualitative study  Community setting | Women aged between 20 and 50  years  N=20 | **Individual perception**:   - Faith in a curative medical system decreases perceived susceptibility. - Women believed that the doctor would diagnose what was happening inside their body   **Modifying factors:**   - Lack of knowledge about cervical cancer and cancer screening was negatively associated to the poor screening uptake for cervical cancer. - Women thought that screening was only performed when the symptoms of cervical cancer appeared.   **Cultural factors increase perceived threat:**   - Shame of being “sick” reduces the perceived threat of cervical cancer screening. - Lack of trust and privacy was found to be associated with screening uptake.   **Perceived barriers:**   - Fatalistic view of cervical cancer increases perceived barriers - Women will think cervical cancer was a chronic and incurable diseases and they believed that the screening would not change the disease condition. - Women was afraid of being diagnosed with cervical cancer and dying soon from diseases as a result they did not undergo screening. - Time increases perceived barriers - Lack of time was found to be associated with screening uptake among the women. - Health care facility navigation increases perceive barriers - Poor knowledge about the healthcare facility was also associated with screening uptake. - Fear of hidden costs increases perceived barriers - Transportation cost and treatment expenses were found to be associated with uptake of screening test.   **Cues to action:**   - Personal experiences with family members with cervical cancer does not increase perceived threat - Chronic abdominal pain as a cue to action - Case study of an outlier: experience with HIV and early intervention as a cue to action |
| Darj et al. 2019  Nepal | Qualitative study  (Focus group discussion and in-depth interview)  Hospital/ Community setting | Women aged between 25 and 60 years  N=72 | **Misconception about cervical cancer**   - Women thought that cervical cancer was dangerous, unusual, and required surgery. - Lack of awareness of the symptoms and so they sought healthcare only when physical symptoms appeared - Women did not link their scare knowledge of the disease to risk factors or possible causes of cervical cancer such as HPV and no awareness of HPV screening, as a means of early detection of precancerous stages and possible preventive treatment to avoid cancer development. - Women associated going to hospital with the experience of certain physical symptoms, such as abdominal pain, infections, or other complications caused by cervical cancer. If they did not have any kind of symptoms, experience difficulties during sexual intercourse, or have any abnormal discharge, they did not feel any need to seek healthcare.   **Misconception about screening procedure**   - Women who had participated in an awareness programme mistakenly believed that screening should be completed every six months and should attend their nearest health centre for screening. - They believed that cervical cancer could be screened and treated simultaneously, and some women mistakenly believed that cervical screening was used to detect other uterine complications. - Some women believed that if they had a negative result, they would never ‘catch’ cervical cancer. - Some women perceived that anxiety and fear about the result could cause women to avoid attending screening health posts.   **Barriers**  **Sociocultural barriers**   - Women thought that other community members would become suspicious and gossip about them if they attended clinics for screening. Some women feared that if they attended a clinic for cervical cancer screening, it might be assumed that they had committed adultery. - Women lacked decision making power and felt restricted about talking and expressing their feelings. They were dependent upon decisions about their health being made by their husbands or in laws, they might be prevented from attending a screening clinic. - Women were responsible for all households’ duties including cooking for family, cleaning the house, taking care of the children, working in the fields. So, they had no time for themselves, for health screening. - Women felt ashamed to show their genitals to others and so they were hesitant to attend clinics for check-ups even when they experienced negative physical symptoms. - Having male health care workers perform the screenings was perceived a cause of shame.   **Previous experience and encounter with health care provider**   - Lack of trust in healthcare provider (HCP) due to experiences of inappropriate behaviour by some of them. - Service provider’s negligence or incompetence would cause their health to deteriorate and cause complications and therefore they were hesitant about attending clinics.   **Financial barrier**   - Lack of money was considered as a major barrier to women’s attendance at screening clinics. - The women were aware of the cost of screening procedures, and they concerned about incurring unexpected costs if additional investigations and surgery were necessary. - Women stopped attending for screening after finding their test results were negative as they considered future screening would be a waste of money. - Government do not provide subsidy for screening services. Women concerned about the financial barrier to the screening uptake, they could go for free screening provided by the free health check-ups camps, but they couldn’t go for further treatment in case of referral due to financial barrier.   **Geographical challenges**   - Those lived in rural areas were less likely to access screening services because many village health posts lacked screening facilities. - The nearest health post providing a cervical cancer screening services could be a long distance from their homes, and they need to walk through physically challenging conditions on slippery roads in a hilly district. - The access could be improved by setting up mobile screening camps or making the service available in health centres nearer to where they lived.   **Facilitator: awareness programme**   - Women-friendly awareness programme targeting cervical cancer screening would increase their knowledge and motivate them to use screening services. - These programmes could be held by organisations or information can be given through news programmes on TV, radio and advertisement.   **Facilitator: enhancing motivation**   - Family support and permission to attend for screening was important to motivate women to attending screening. - Gaining knowledge from others such as women’s group where they share problems and information openly would motivate them to attend screening. |

Note. Abbreviations: OR= odd ratio, AOR= adjusted odd ratio, CI= confidence interval, STDs= sexually transmitted diseases, MSPs = multiple sexual partners.

**S2 Table. Summary of the included studies**
