# Supplementary material for: Determinants of cervical cancer screening utilisation among women in the least developed countries: A systematic review and meta-analysis
Source: PLoS One. 2025 Jun 24;20(6):e0321627. doi: 10.1371/journal.pone.0321627 (PMC12186883; doi:10.1371/journal.pone.0321627)
Supplement: S4 Table — (DOC) [file pone.0321627.s004.doc]

**S4 Table: List of full-text excluded articles (titles and reasons)**

**Reasons for exclusion: 1 Irrelevant outcome; 2 Irrelevant participants; 3 Irrelevant study setting; 4 Irrelevant study design & 5 Irrelevant age group**

| **Number** | **Title** | **Resources** | **Reasons for exclusion** |
| --- | --- | --- | --- |
| 1 | Cervical cancer screening “see and treat approach”: real-life uptake after invitation  and associated factors at health facilities in Gondar, Northwest Ethiopia | BMC Cancer 2021 | 1 |
| 2 | Barriers to follow-up after an abnormal cervical cancer screening result and the role of male partners: a qualitative study | BMJ Open, 2021 | 1 |
| 3 | Factors associated with a cervical high-grade lesion on cytology or a positive visual inspection with acetic acid among more than 3300 Tanzanian women | Tropical Medicine and International Health, 2019 | 1 |
| 4 | Uptake of pre-cervical cancer screening and associated factors among reproductive age women in Debre Markos town, Northwest Ethiopia, 2017 | BMC Public Health, 2019 | 5 |
| 5 | Comprehensive Knowledge towards Cervical Cancer and Associated Factors among Women in Durame Town, Southern Ethiopia | Journal of Cancer Epidemiology, 2020 | 1 |
| 6 | Exploring Complicity of Cervical Cancer Screening in Malawi: The Interplay of  Behavioral, Cultural, and Societal Influences | Asia Pacific Journal of Oncology Nursing, 2019 | 1 |
| 7 | Barriers and Facilitators to Cervical Cancer Screening, Diagnosis, Follow-Up Care and Treatment: Perspectives of Human Immunodeficiency Virus-PositiveWomen and Health Care  Practitioners in Tanzania | Global Health and Cancer, 2019 | 2 |
| 8 | Motivations and barriers to cervical cancer screening among HIV infected women in  HIV care: a qualitative study | BMC Women’s Health, 2015 | 2 |
| 9 | Knowledge and practice of cervical cancer screening and associated factors among  reproductive age group women in districts of Gurage zone, Southern Ethiopia. A cross-sectional study | Plos One, 2020 | 5 |
| 10 | Barriers to Cervical Cancer Screening Among Haitian  Immigrant Women in Little Haiti, Miami | Journal of cancer education: the official journal of the American Association for Cancer Education, 2010 | 1 |
| 11 | Factors associated with cervical cancer screening participation among migrant  women in Europe: a scoping review | International Journal for Equity in Health, 2020 | 4 |
| 12 | Psychographic predictors of intention to use cervical cancer screening services among women attending maternal and child health services in Southern Ethiopia: the theory of planned behavior (TPB) perspective | BMC Public Health,2021 | 1 |
| 13 | Knowledge of cervical cancer screening and associated factors among women attending maternal health services at Aira Hospital, West Wollega, Ethiopia | Sage Open Medicine, 2021 | 1 |
| 14 | Prevalence and determinants of cervical cancer screening in five sub-Saharan African countries: A population-based study | Cancer Epidemiology, 2021 | 3 |
| 15 | Barriers affecting uptake of cervical cancer screening in low and middle income countries: A systematic review | Indian Journal of Cancer, 2018 | 4 |
| 16 | Factors affecting cervical cancer screening uptake, visual inspection with acetic acid  positivity and its predictors among women attending cervical cancer screening service in Addis Ababa, Ethiopia | BMC Women’s Health, 2020 | 1 |
| 17 | Factors Associated with Late Diagnosis of Cervical Cancer in Nepal | Asian Pacific Journal of Cancer Prevention | 1 |
| 18 | Factors associated with high-risk human papillomavirus test utilization and infection:  a population-based study of uninsured and underinsured women | BMC Women’s Health, 2018 | 1 |
| 19 | Health systems challenges in cervical cancer prevention program in Malawi | Global Health Action, 2015 | 1 |
| 20 | Cervical Cancer Screening, Adherence to and Challenges of Follow-Up in Resources  Poor Setting | Open Journal of Preventive Medicine, 2019 | 1 |
| 21 | Factors Associated with Uptake of Visual Inspection with Acetic Acid (VIA) for Cervical  Cancer Screening in Western Kenya | Plos One, 2016 | 1 |
| 22 | Health seeking behavior and its determinants for cervical cancer among women of childbearing age in Hossana Town, Hadiya zone, Southern Ethiopia: community based cross sectional study | BMC Cancer, 2018 | 1 |
| 23 | Comprehensive Knowledge towards Cervical Cancer and Associated Factors among Women in Durame Town, Southern Ethiopia | Journal of Cancer Epidemiology, 2020 | 1 |
| 24 | Knowledge about cervical cancer and barriers toward cervical cancer screening among HIV-positive women attending public health centers in Addis Ababa city, Ethiopia | Cancer Medicine, 2018 | 2 |
| 25 | Knowledge Toward Cervical Cancer and Its Determinants Among Women Aged 30-49 in Jimma Town, Southwest Ethiopia | Cancer Control: Journal of the Moffit Cancer Centre, 2020 | 1 |
| 26 | Factors influencing the uptake of cervical cancer screening services in Tanzania: A health system perspective from national and district levels | Nursing One, 2019 | 1 |
| 27 | Health systems challenges in cervical cancer prevention program in Malawi | Global Health Action, 2015 | 1 |
| 28 | Risk factors for VIA positivity and determinants of screening attendances in Dar es Salaam, Tanzania | BMC Public Health, 2012 | 1 |
| 29 | Acceptance of peer navigators to reduce barriers to cervical cancer screening and treatment among women with HIV infection in Tanzania | International journal of gynaecology and obstetrics: the official organ of the International Federation of Gynaecology and Obstetrics, 2017 | 2 |
| 30 | Acceptability of cervical cancer screening using visual inspection among women attending a childhood immunization clinic in Uganda | Papillomavirus research (Amsterdam, Netherlands), 2017 | 1 |
| 31 | Health professionals' willingness to pay and associated factors for cervical cancer screening program at College of Medicine and Health Sciences, University of Gondar, Northwest Ethiopia | PloS one, 2019 | 1 |
| 32 | Predictors of cervical cancer screening practice among HIV positive women attending adult anti-retroviral treatment clinics in Bishoftu town, Ethiopia: the application of a health belief model | BMC cancer, 2019 | 2 |
| 33 | Factors affecting the practices of cervical cancer screening among female nurses at public health institutions in mekelle town, northern Ethiopia, 2014: A cross-sectional study | Journal of Cancer Research, 2016 | 1 |
| 34 | Barriers and Facilitators to Cervical Cancer Screening, Diagnosis, Follow‐Up Care and Treatment: Perspectives of Human Immunodeficiency Virus‐Positive Women and Health Care Practitioners in Tanzania | The Oncologist, 2019 | 2 |
| 35 | Predictors of Precancerous Cervical Lesions Among Women Screened for Cervical Cancer in Bahir Dar Town, Ethiopia: A Case-Control Study | Cancer management and research, 2020 | 4 |
| 36 | Factors affecting utilization of cervical cancer screening services among women attending public hospitals in Tigray region, Ethiopia, 2018; Case control study | Plos one, 2019 | 4 |
| 37 | Knowledge, attitude and practice of cervical cancer screening and associated factors amongst female students at Wollega University, western Ethiopia | BMC Research Notes, 2019 | 5 |
