# Supplementary material for: Determinants of cervical cancer screening utilisation among women in the least developed countries: A systematic review and meta-analysis
Source: PLoS One. 2025 Jun 24;20(6):e0321627. doi: 10.1371/journal.pone.0321627 (PMC12186883; doi:10.1371/journal.pone.0321627)
Supplement: S5 Table — (DOCX) [file pone.0321627.s005.docx]

**S5 Table: All the included studies identified during the literature search (after duplicate studies were removed)**

**(Note: Reasons for exclusion of study: 1=Irrelevant title; 2= Irrelevant outcome; 3=Irrelevant participants; 4= Irrelevant study setting; 5= Irrelevant study design, 6= Irrelevant age group; 7= Conference paper/ abstract)**

| **S.N** | **All the studies** | **Included** | **Excluded** | **Reasons for exclusion** |
| --- | --- | --- | --- | --- |
|  | Cervical cancer control in developing countries: memorandum from a WHO meeting |  | Yes | 1 |
|  | Canadian Society for Epidemiology and Biostatistics 2013 National Student Conference, CSEB 2013 |  | Yes | 1 |
|  | 3rd Regional International Gynecologic Cancer Society, IGCS Meeting 2014 |  | Yes | 7 |
|  | IDSOG Abstracts 2015 |  | Yes | 7 |
|  | Poster Abstracts from the 7th Annual CUGH Conference: Bridging to a Sustainable Future in Global Health |  | Yes | 7 |
|  | Screening for cervical cancer in Senegal: Contributing factors |  | Yes | 2 |
|  | Psychographic predictors of intention to use cervical cancer screening services among women attending maternal and child health services in Southern Ethiopia: the theory of planned behavior |  | Yes | 2 |
|  | Detection of Oncoprotein by a Novel Immunochromatoghaphic Test Depending on Age and Parity of the Patients Attending at Mymensingh Medical College Hospital, Bangladesh |  | Yes | 1 |
|  | Demand for Cervical Cancer Screening in Tigray Region of Ethiopia in 2018: A Community-Based Cross-Sectional Study |  | Yes | 1 |
|  | Cervical cancer screening behavior and associated factors among women of Ugrachandi Nala, Kavre, Nepal | Yes | | |
|  | A clinical breast screening pilot study in Malawi |  | Yes | 5 |
|  | Single-visit approach: An ethiopian case study |  | Yes | 5 |
|  | Cervical cancer risk factors and screening preferences among Muslim women in Monrovia, Liberia |  | Yes | 3 |
|  | Knowledge and practice of Togolese women concerning cervical cancer |  | Yes | 3 |
|  | Working with organized groups to change cultural beliefs and norms toward cancer in Uganda |  | Yes | 1 |
|  | Socio-economic and health access determinants of breast and cervical cancer screening in low-income countries: analysis of the World Health Survey | Yes | | |
|  | Awareness about cervical cancer and its prevention among the female respondents of general hospital in bangladesh |  | Yes | 1 |
|  | Predictors of Precancerous Cervical Lesions Among Women Screened for Cervical Cancer in Bahir Dar Town, Ethiopia: A Case-Control Study |  | Yes | 5 |
|  | Intention to Screen for Cervical Cancer Among Child Bearing Age Women in Bahir Dar City, North-West Ethiopia: Using Theory of Planned Behavior |  | Yes | 1 |
|  | Factors affecting attendance to cervical cancer screening among women in the Paracentral Region of El Salvador: a nested study within the CAPE HPV screening program |  | Yes | 3 |
|  | Knowledge, attitude and practice about cancer of the uterine cervix among women living in Kinshasa, the Democratic Republic of Congo |  | Yes | 1 |
|  | Cervical samples dried on filter paper and dried vaginal tampons can be useful to investigate the circulation of high-risk HPV in Congo |  | Yes | 1 |
|  | Knowledge, attitude and practice about cancer of the uterine cervix among women living in Kinshasa, the Democratic Republic of Congo |  | Yes | 1 |
|  | Knowledge, Attitudes and Practices of Sudanese Women Regarding the Pap Smear Test and Cervical Cancer |  | Yes | 1 |
|  | Knowledge of cervical cancer screening and associated factors among women attending maternal health services at Aira Hospital, West Wollega, Ethiopia |  | Yes | 2 |
|  | Implementing visual cervical cancer screening in Senegal: A cross-sectional study of risk factors and prevalence highlighting service utilization barriers |  | Yes | 1 |
|  | Cervical cancer in Haiti, a major public health problem: A retrospective and descriptive study on cervical cancer biopsies carried out in the capital's laboratories from 2013 to 2015 |  | Yes | 7 |
|  | Cervical cancer in Bangladesh: community perceptions of cervical cancer and cervical cancer screening |  | Yes | 1 |
|  | Knowledge towards cervical cancer screening and associated factors among urban health extension workers at Addis Ababa, Ethiopia: facility based cross-sectional survey |  | Yes | 2 |
|  | Worldwide burden of cervical cancer in 2008 |  | Yes | 1 |
|  | Estimates of incidence and mortality of cervical cancer in 2018: a worldwide analysis |  | Yes | 1 |
|  | Incidence and mortality of cervical cancer in Latin America |  | Yes | 1 |
|  | Jordanian women's attitudes towards cervical cancer screening: has the pattern changed? |  | Yes | 3 |
|  | Factors associated with high-risk human papillomavirus test utilization and infection: a population-based study of uninsured and underinsured women |  | Yes | 2 |
|  | Cervical cancer screening service utilization and associated factors among HIV positive women attending adult ART clinic in public health facilities, Hawassa town, Ethiopia: a cross-sectional study |  | Yes | 1 |
|  | High prevalence of cervical squamous intraepithelial lesions in women on antiretroviral therapy in Cameroon: Is targeted screening feasible? |  | Yes | 1 |
|  | Study on risk factors for cervical carcinoma at Central Womens Hospital, Yangon, Myanmar |  | Yes | 7 |
|  | Knowledge, attitude and practice for cervical cancer prevention and control among women of childbearing age in Hossana Town, Hadiya zone, Southern Ethiopia: Community-based cross-sectional study |  | Yes | 1 |
|  | Uptake of cervical cancer screening service and associated factors among age-eligible women in Ethiopia: systematic review and meta-analysis |  | Yes | 5 |
|  | Utilization of cervical cancer screening and associated factors among women in Debremarkos town, Amhara region, Northwest Ethiopia: Community based cross-sectional study | Yes | | |
|  | Prevalence and determinants of breast cancer screening in four low and middle income countriescountries: a population-based study |  | Yes | 4 |
|  | Barriers to follow-up after an abnormal cervical cancer screening result and the role of male partners: a qualitative study |  | Yes | 2 |
|  | Reproducibility of a Rapid Human Papillomavirus Test at Different Levels of the Healthcare System in Tanzania: The AISHA Study |  | Yes | 1 |
|  | Some Traits on the Outcome of the Treatment of Cervical Cancer in Tanzania: A Case Study of Ocean Road Cancer Institute (ORCI) |  | Yes | 1 |
|  | Cost-utility of HPV for prevention of cervical cancer in the roraima (Brazilian amzonic region): A Markov model approach |  | Yes | 7 |
|  | Factors associated with a cervical high-grade lesion on cytology or a positive visual inspection with acetic acid among more than 3300 Tanzanian women |  | Yes | 2 |
|  | Investigating Bangladeshi Rural Women's Awareness and Knowledge of Cervical Cancer and Attitude Towards HPV Vaccination: a Community-Based Cross-Sectional Analysis |  | Yes | 1 |
|  | Uptake of pre-cervical cancer screening and associated factors among reproductive age women in Debre Markos town, Northwest Ethiopia, 2017 |  | Yes | 2 |
|  | Universal routine HPV vaccination for young girls in Uganda: a review of opportunities and potential obstacles |  | Yes | 1 |
|  | Epidemiology of HPV genotypes in Uganda and the role of the current preventive vaccines: A systematic review |  | Yes | 5 |
|  | Epidemiology of HPV genotypes in Uganda and the role of the current preventive vaccines: A systematic review |  | Yes | 5 |
|  | Assessing the acceptability of self-sampling for HPV among Haitian immigrant women: CBPR in action |  | Yes | 1 |
|  | Barriers and Facilitators to Cervical Cancer Screening, Diagnosis, Follow‐Up Care and Treatment: Perspectives of Human Immunodeficiency Virus‐Positive Women and Health Care Practitioners in Tanzania |  | Yes | 3 |
|  | Cervical Cancer Screening Service Uptake and Associated Factors among Age Eligible Women in Mekelle Zone, Northern Ethiopia, 2015: A Community Based Study Using Health Belief Model | Yes | | |
|  | Incidence of human papillomavirus, cervical dysplasia and cervical cancer among women in Liberia: Assessing the burden of disease |  | Yes | 1 |
|  | Cervical cancer in Ethiopia – predictors of advanced stage and prolonged time to diagnosis |  | Yes | 1 |
|  | Detection of Human Papilloma virus by Molecular method from Patients Attending at Colposcopy Clinic of Mymensingh Medical College Hospital, Mymensingh |  | Yes | 1 |
|  | Knowledge, Attitude and Practice of Bangladeshi Women towards Breast Cancer: A Cross Sectional Study |  | Yes | 1 |
|  | Comprehensive knowledge and uptake of cervical cancer screening is low among women living with HIV/AIDS: The case of northwestern ethiopia |  | Yes | 7 |
|  | Cervical Cancer Screening Utilization and Associated Factors Among Women Aged 30 to 49 Years in Dire Dawa, Eastern Ethiopia | Yes | | |
|  | Precancerous cervical lesion and associated factors among HIV-infected women on ART in Amhara Regional State, Ethiopia: A hospital-based cross-sectional study |  | Yes | 1 |
|  | Epidemiological profile and factors associated with mortality among women with gynecological cancers in a Haitian cancer program |  | Yes | 7 |
|  | Implementation of a human papillomavirus screen-and-treat model in Mwanza, Tanzania: training local healthcare workers for sustainable impact |  | Yes | 1 |
|  | Developing sustainable cervical cancer screening in mwanza, tanzania: Implementation of a screen-and-treat model |  | Yes | 7 |
|  | Cervical cancer screening and treatment on the dominican republic/Haitian border |  | Yes | 7 |
|  | Risk Factors Associated with Invasive Cervical Carcinoma among Women Attending Jimma University Specialized Hospital, Southwest Ethiopia: A Case Control Study |  | Yes | 1 |
|  | Program organization rather than choice of test determines success of cervical cancer screening: Case studies from Bangladesh and India |  | Yes | 5 |
|  | Barriers and drivers of cervical cancer screening in Gonaives, Haiti |  | Yes | 7 |
|  | Eliminating Deaths From Cervical Cancer-Report of a Panel at the 7th Annual Symposium on Global Cancer Research, a Satellite Meeting at the Consortium of Universities for Global Health 10th Annual Meeting |  | Yes | 1 |
|  | Integration of comprehensive women's health programmes into health systems: cervical cancer prevention, care and control in Rwanda |  | Yes | 1 |
|  | Barriers and facilitators to uptake of cervical cancer screening among women in Uganda: a systematic review |  | Yes | 5 |
|  | Cervical cancer screening in a slum setting using visual inspection with acetic acid: A pilot study |  | Yes | 7 |
|  | Cervical cancer screening uptake in women aged between 15 and 64 years in Mozambique |  | Yes | 1 |
|  | Genital self-sampling for HPV-based cervical cancer screening: a qualitative study of preferences and barriers in rural Ethiopia |  | Yes | 1 |
|  | Contraceptive camps as a means for Ugandan health workers to gain practical experience in delivering family planning and cervical cancer screening |  | Yes | 7 |
|  | Acceptability of self-collected vaginal samples for HPV testing in an urban and rural population of Madagascar |  | Yes | 1 |
|  | Motivations and barriers to cervical cancer screening among HIV infected women in HIV care: a qualitative study |  | Yes | 3 |
|  | Screening using self-collected samples |  | Yes | 7 |
|  | Knowledge regarding cervical cancer among undergraduate female students at a selected college of Lalitpur, Nepal |  | Yes | 1 |
|  | Different routes, similar destination: Building breast care models in Tanzania, Zambia, and Colombia |  | Yes | 7 |
|  | Pilot evaluation of cervical cancer screening modalities in a low resource Ugandan setting |  | Yes | 7 |
|  | Prevalence of HPV 16/18 amongst medical students at the University A. Neto |  | Yes | 7 |
|  | Use of thermocoagulation within a 'screen and treat' cervical cancer screening programme in malawi: Outcomes at one year, professional perspectives, and client experience |  | Yes | 7 |
|  | Cervical cancer screening in low-resource settings: A cost-effectiveness framework for valuing tradeoffs between test performance and program coverage |  | Yes | 1 |
|  | Evidence-based policy choices for efficient and equitable cervical cancer screening programs in low-resource settings |  | Yes | 1 |
|  | A randomized trial ofa community health worker led intervention using hpv self-sampling to increase cervical cancer screening among minority women: Preliminary findings |  | Yes | 7 |
|  | HPV infection and cervical disease: a review |  | Yes | 5 |
|  | Assessing the Acceptability, Feasibility, and Effectiveness of a Tablet-Based Cervical Cancer Educational Intervention |  | Yes | 1 |
|  | Characteristics associated with regular cervical cancer screening in tigray, ethiopia |  | Yes | 7 |
|  | Screening and cancer prevention in underdeveloped environments: A global view |  | Yes | 7 |
|  | A survey of knowledge and attitudes relating to cervical and breast cancer among women in Ethiopia |  | Yes | 1 |
|  | 94% Population HIV testing coverage with repeat hybrid mobile testing in East Africa |  | Yes | 7 |
|  | Human Papillomavirus Infection and Cervical Cancer: Epidemiology, Screening, and Vaccination—Review of Current Perspectives |  | Yes | 1 |
|  | Perceived benefits and barriers towards cervical cancer screening among women >15 years in Arsi zone, Southeastern Ethiopia: Application of the Health Belief Model (HBM) in a community- based cross-sectional study |  | Yes | 7 |
|  | Introduction of HPV test for cervical cancer screening in Cambodia as low resource setting |  | Yes | 7 |
|  | Uptake of a community-based screen-and-treat cervical cancer prevention strategy in rural malawi |  | Yes | 7 |
|  | Uptake and safety of community-based "screen-and-treat" with thermal ablation preventive therapy for cervical cancer prevention in rural Lilongwe, Malawi |  | Yes | 1 |
|  | Reducing the global cancer Burden - Surveillance and control one country at a time |  | Yes | 7 |
|  | Barriers to Cervical Cancer Screening in Burkina Faso: Needs for Patient and Professional Education | Yes | | |
|  | Implementation of human papillomavirus video education for women participating in mass cervical cancer screening in Tanzania |  | Yes | 7 |
|  | Implementation of human papillomavirus video education for women participating in mass cervical cancer screening in Tanzania |  | Yes | 1 |
|  | Feasibility Of Visual Inspection With Acetic Acid (VIA) Screening For Cervical Cancer In Tanzania With Emphasis On Baseline Knowledge And Educational Intervention |  | Yes | 7 |
|  | Cervical human papillomavirus infection among young women engaged in sex work in Phnom Penh, Cambodia: prevalence, genotypes, risk factors and association with HIV infection |  | Yes | 5 |
|  | Cervical cancer screening - The challenges of complete pathways of care in low-income countries: Focus on Malawi |  | Yes | 7 |
|  | Promoting HPV vaccination among adolescent girls in Ghana, West Africa: A case study |  | Yes | 7 |
|  | Cervical cancer screening in rural East Africa |  | Yes | 7 |
|  | Cervical cancer screening and HPV vaccine acceptability among rural and urban women in Kilimanjaro Region, Tanzania |  | Yes | 1 |
|  | Barriers and facilitators to cervical cancer screening in Nepal: A qualitative study | Yes | | |
|  | Involving men in cervical cancer prevention: A qualitative enquiry into male perspectives on screening and HPV vaccination in mid-western Uganda |  | Yes | 7 |
|  | Women's cancer screening in haiti: Increasing access by bringing services to the workplace |  | Yes | 7 |
|  | Prevalence and factors associated with VIA positive result among clients screened at Family Guidance Association of Ethiopia, south west area office, Jimma model clinic, Jimma, Ethiopia 2013: a cross-sectional study |  | Yes | 1 |
|  | Case Study: Schistosomiasis of the Endocervical Canal, A Rarely Reported Localization |  | Yes | 5 |
|  | Poly(ADP-ribose)polymerase1: A potential molecular marker to identify cancer during colposcopy procedures |  | Yes | 1 |
|  | Prevention of cervical cancer in developing countries |  | Yes | 1 |
|  | The prevention of cervical cancer in developing countries |  | Yes | 5 |
|  | Cytological screening for cervical cancer prevention |  | Yes | 7 |
|  | Low level of knowledge about cervical cancer among Ethiopian women: a systematic review and meta-analysis |  | Yes | 5 |
|  | Factors associated with advanced stage at diagnosis of cervical cancer in Addis Ababa, Ethiopia: a population-based study |  | Yes | 1 |
|  | Peace corps partnering for health services implementation research: Volunteer perspectives |  | Yes | 7 |
|  | Visual inspection with acetic acid and Lugol's iodine in cervical cancer screening at the general referral hospital Kayembe in Mbuji-Mayi, Democratic Republic of Congo |  | Yes | 1 |
|  | Cervical Cancer Screening Uptake and Associated Factors among HIV-Positive Women in Ethiopia: A Systematic Review and Meta-Analysis |  | Yes | 5 |
|  | Barriers affecting uptake of cervical cancer screening in low and middle income countries: A systematic review |  | Yes | 5 |
|  | The Association of Molecular Biomarkers in the Diagnosis of Cervical Pre-Cancer and Cancer and Risk Factors in Senegalese |  | Yes | 1 |
|  | Systematic screening for cervical cancer in Dakar region: prevalence and correlation with biological and socio-demographic parameters |  | Yes | 1 |
|  | Mass screening campaigns to increase awareness of cervical cancer treatment and prevention in Equatorial Guinea |  | Yes | 7 |
|  | Socio-demographic and clinical characteristic of women availing pap smear services in Samtse District, Bhutan |  | Yes | 1 |
|  | The Role of Nurse Practitioners in Global Cancer Care |  | Yes | 7 |
|  | Female urogenital schistosomiasis in Tanzania's lake zone region: A highly-sporadic distribution among women in eight rural villages |  | Yes | 7 |
|  | Knowledge about cervical cancer screening and its practice among female health care workers in southern Ethiopia: a cross-sectional study |  | Yes | 1 |
|  | [Cervical cancer screening in rural Madagascar: Feasibility, coverage and incidence] |  | Yes | 1 |
|  | The prevalence and risk factors of cervical dysplasia and cervical cancer in the Kedougou Region, Senegal |  | Yes | 7 |
|  | Implementing visual cervical cancer screening in Senegal: a cross-sectional study of risk factors and prevalence highlighting service utilization barriers |  | Yes | 1 |
|  | Implementation of a high-dose-rate brachytherapy program for carcinoma of the cervix in Senegal: a pragmatic model for the developing world |  | Yes | 1 |
|  | Awareness of female health workers and non health workers on cervical cancer and cervical cancer screening: South-South, Nigeria |  | Yes | 7 |
|  | Knowledge and practice of cervical cancer screening and associated factors among reproductive age group women in districts of Gurage zone, Southern Ethiopia. A cross-sectional study |  | Yes | 6 |
|  | Comprehensive knowledge and uptake of cervical cancer screening is low among women living with HIV/AIDS in Northwest Ethiopia |  | Yes | 1 |
|  | SMS behaviour change communication and eVoucher interventions to increase uptake of cervical cancer screening in the Kilimanjaro and Arusha regions of Tanzania: a randomised, double-blind, controlled trial of effectiveness |  | Yes | 1 |
|  | Generating evidence on new screening strategies for cervical cancer in low-resource areas |  | Yes | 1 |
|  | Factors influencing Malawian women's willingness to self-collect samples for human papillomavirus testing |  | Yes | 5 |
|  | Cervical Cancer Screening Acceptance among Women in Dabat District, Northwest Ethiopia, 2017: An Institution-Based Cross-Sectional Study |  | Yes | 1 |
|  | Accuracy of the Triple Test Versus Colposcopy for the Diagnosis of Premalignant and Malignant Cervical Lesions |  | Yes | 1 |
|  | Factors affecting cervical cancer screening uptake, visual inspection with acetic acid positivity and its predictors among women attending cervical cancer screening service in Addis Ababa, Ethiopia |  | Yes | 2 |
|  | Frequency and pattern of gynecologic cancers from 2010 to 2014 in Beira, Mozambique |  | Yes | 1 |
|  | Prevalence of precancerous cervical lesions in pregnancy using visual inspection with acetic acid |  | Yes | 7 |
|  | Is screen-and-treat approach suited for screening and management of precancerous cervical lesions in Sub-Saharan Africa? |  | Yes | 1 |
|  | Barriers to cervical cancer screening in Mulanje, Malawi: a qualitative study | Yes | | |
|  | Cervical Cancer |  | Yes | 5 |
|  | The IARC commitment to cancer prevention: the example of papillomavirus and cervical cancer |  | Yes | 1 |
|  | Vaccination against human papillomavirus infection: A new paradigm in cervical cancer control |  | Yes | 7 |
|  | Vaccination against human papillomavirus infection: a new paradigm in cervical cancer control |  | Yes | 1 |
|  | Integrating human papillomavirus vaccination in cervical cancer control programmes |  | Yes | 1 |
|  | HPV Testing from Dried Urine Spots as a Tool for Cervical Cancer Screening in Low-Income Countries |  | Yes | 1 |
|  | Cervical cancer prevention in Senegal: an International Cooperation Project Report |  | Yes | 1 |
|  | Inequalities in cervical cancer screening utilisation and results: A comparison between Italian natives and immigrants from disadvantaged countries |  | Yes | 1 |
|  | Human papillomavirus and cervical cancer in Australasia and Oceania: risk-factors, epidemiology and prevention |  | Yes | 1 |
|  | Cervical cancer burden and prevention strategies: Asia Oceania perspective |  | Yes | 1 |
|  | Factors affecting the practices of cervical cancer screening among female nurses at public health institutions in mekelle town, northern Ethiopia, 2014: A cross-sectional study |  | Yes | 2 |
|  | The prevalence of precancerous cervical cancer lesion among HIV-infected women in southern Ethiopia: a cross-sectional study |  | Yes | 1 |
|  | Prevalence and Predictor of Cervical Cancer Screening Service Uptake Among Women Aged 25 Years and Above in Sidama Zone, Southern Ethiopia, Using Health Belief Model | Yes | |  |
|  | Comprehensive knowledge on cervical cancer, attitude towards its screening and associated factors among women aged 30-49 years in Finote Selam town, northwest Ethiopia |  | Yes | 5 |
|  | Cervical cancer screening knowledge and barriers among women in Addis Ababa, Ethiopia |  | Yes | 5 |
|  | Assessment of cervical cancer services and cervical cancer related knowledge of health service providers in public health facilities in Addis Ababa, Ethiopia |  | Yes | 1 |
|  | Comprehensive knowledge about cervical cancer is low among women in Northwest Ethiopia |  | Yes | 1 |
|  | Intention to Screen for Cervical Cancer in Debre Berhan Town, Amhara Regional State, Ethiopia: Application of Theory of Planned Behavior |  | Yes | 1 |
|  | Prevalence and predictors of Pap smear cervical epithelial cell abnormality among HIV-positive and negative women attending gynecological examination in cervical cancer screening center at Debre Markos referral hospital, East Gojjam, Northwest Ethiopia |  | Yes | 1 |
|  | Reasons for Not Attending Cervical Cancer Screening and Associated Factors in Rural Ethiopia |  | Yes | 2 |
|  | A public health approach to cervical cancer control: considerations of screening and vaccination strategies |  | Yes | 1 |
|  | CHAPTER 10 A public health approach to cervical cancer control: Considerations of screening and vaccination strategies |  | Yes | 1 |
|  | Projected cervical Cancer incidence in Swaziland using three methods and local survey estimates |  | Yes | 1 |
|  | Determining the optimal cervical carcinoma screening method in HIV positive and HIV negative cambodian women |  | Yes | 7 |
|  | PCR-RFLP assay as an option for primary HPV test |  | Yes | 1 |
|  | Development of a cervical cancer screening protocol in the korile slum area of Dhaka, Bangladesh |  | Yes | 7 |
|  | Cervical cytology in Zambia |  | Yes | 2 |
|  | An approach to establish behavioral determinants for predicting participation in cervical cancer screening of Haitian women |  | Yes | 7 |
|  | Barriers and facilitators to cervical cancer screening uptake among women in Nepal – a qualitative study | Yes | | |
|  | HIV/HPV co-infection: Monitoring and treatment in sub-saharan africa: A public health model intervention |  | Yes | 7 |
|  | Factors associated with late diagnosis of cervical cancer in Nepal |  | Yes | 2 |
|  | Health seeking behavior and its determinants for cervical cancer among women of childbearing age in Hossana Town, Hadiya zone, Southern Ethiopia: community based cross sectional study |  | Yes | 2 |
|  | Magnitude and associated factors of VIA positive test results for cervical cancer screening among refugee women aged 25-49 years in North Ethiopia |  | Yes | 2 |
|  | Patient side cost and its predictors for cervical cancer in Ethiopia: a cross sectional hospital based study |  | Yes | 2 |
|  | Descriptive Epidemiology of breast and gynecological cancers among patients attending Saint Paul's Hospital Millennium Medical College, Ethiopia |  | Yes | 1 |
|  | Breaking the silence |  | Yes | 7 |
|  | Higher educational attainment associated with reduced likelihood of abnormal cervical lesions among Zambian women - a cross sectional study |  | Yes | 1 |
|  | Influence of HIV-1 and/or HIV-2 infection and CD4 count on cervical HPV DNA detection in women from Senegal, West Africa |  | Yes | 1 |
|  | Delay to diagnosis for cervical cancer among Rwandan women |  | Yes | 7 |
|  | Eliminating Cervical Cancer in Mali and Senegal, Two Sub-Saharan Countries: Insights and Optimizing Solutions |  | Yes | 1 |
|  | The UICC Cervical Cancer Initiative: A comprehensive program for cervical cancer prevention worldwide including a fellowship program |  | Yes | 7 |
|  | The UICC Cervical Cancer Initiative: A comprehensive program for cervical cancer prevention worldwide including a fellowship program |  | Yes | 1 |
|  | Beliefs, perceptions and health-seeking behaviours in relation to cervical cancer: a qualitative study among women in Uganda following completion of an HPV vaccination campaign |  | Yes | 1 |
|  | Cervical cancer screening among Sudanese women |  | Yes | 7 |
|  | Cervical cancer screening among Sudanese women |  | Yes | 1 |
|  | Scaling up proven innovative cervical cancer screening strategies: Challenges and opportunities in implementation at the population level in low- and lower-middle-income countries |  | Yes | 2 |
|  | Cervical cancer in low-income countries: A Bangladeshi perspective |  | Yes | 4 |
|  | Cancer in Low- and Middle-Income Countries: An Economic Overview |  | Yes | 5 |
|  | Implementation of VIA For Cervical Cancer Screening in a Sexually Transmitted Infection Clinic in Lilongwe, Malawi |  | Yes | 7 |
|  | Cervical cancer prevention in lmics: Are we on the path to elimination? |  | Yes | 7 |
|  | Cervical cancer in low and middle-income countries |  | Yes | 1 |
|  | Cervical cancer prevention in Sudan. Barriers and missed opportunities |  | Yes | 7 |
|  | Cervical cancer screening in primary health care setting in Sudan: a comparative study of visual inspection with acetic acid and Pap smear |  | Yes | 1 |
|  | Predictors of cervical cancer being at an advanced stage at diagnosis in Sudan |  | Yes | 1 |
|  | Predictors of cervical cancer screening uptake in two districts of Central Uganda | Yes | | |
|  | Lack of Understanding of Cervical Cancer and Screening Is the Leading Barrier to Screening Uptake in Women at Midlife in Bangladesh: Population-Based Cross-Sectional Survey | Yes | |  |
|  | Knowledge of cervical cancer and screening barriers among mid-life women in Bangladesh |  | Yes | 7 |
|  | Exploring perceptions of cervical cancer in Dhaka, Bangladesh |  | Yes | 7 |
|  | Bangladesh Midlife Women's Health Study (BMWHS): methods, challenges and experiences |  | Yes | 2 |
|  | Knowledge and awareness of early detection methods and risk factors towards breast and cervical cancer in Bangladesh |  | Yes | 7 |
|  | Awareness towards breast and cervical cancer risk factors and its prevention among students and patients in Bangladesh |  | Yes | 7 |
|  | Precancerous Cervix in Human Immunodeficiency Virus Infected Women Thirty Years Old and above in Northern Uganda |  | Yes | 1 |
|  | Cancer Risk Studies and Priority Areas for Cancer Risk Appraisal in Uganda |  | Yes | 1 |
|  | VIA and rapid HPV testing for cervical cancer screening |  | Yes | 7 |
|  | Generating evidence on new screening strategies for cervical cancer in low-resource areas |  | Yes | 7 |
|  | A scenario of cervical carcinoma in a cancer hospital |  | Yes | 1 |
|  | Effects of human papillomavirus infection with pre-invasive cervical lesions: Bangladesh perspectives |  | Yes | 1 |
|  | Knowledge and use of cervical cancer screening among university female students at Kilimanjaro, Tanzania |  | Yes | 7 |
|  | Cervical cancer screening opportunities for Guinea-Bissau |  | Yes | 1 |
|  | The novel artificial intelligence based paptest cervical cancer image computer aided diagnosis system |  | Yes | 7 |
|  | Acceptance of Cervical Cancer Screening and its Correlates Among Women of a Peri-Urban High-Density Residential Area in Ndola, Zambia |  | Yes | 1 |
|  | Prevalence and predictors of Cervical Intraepithelial Neoplasia among HIV infected women at Bugando Medical Centre, Mwanza-Tanzania |  | Yes | 1 |
|  | Risk factors for VIA positivity and determinants of screening attendances in Dar es Salaam, Tanzania |  | Yes | 2 |
|  | Association between invasive cancer of the cervix and HIV-1 infection in Tanzania: The need for dual screening |  | Yes | 1 |
|  | Knowledge of health care providers on cancer in Magu District, Mwanza Tanzania |  | Yes | 7 |
|  | Community-based cervical cancer screening in Kafue District, Zambia |  | Yes | 7 |
|  | The cooperation between professional societies contributes to the capacity building and system development for prevention and control of cancer in low- and middle-income countries: the practice of Cervical Cancer Prevention and Control Project in Cambodia |  | Yes | 1 |
|  | Innovative approaches to promoting cervical health and raising cervical cancer awareness by use of existing cultural structures in resource-limited countries: experiences with traditional marriage counseling in Zambia |  | Yes | 1 |
|  | Implementation and Operational Research: Age Distribution and Determinants of Invasive Cervical Cancer in a "Screen-and-Treat" Program Integrated With HIV/AIDS Care in Zambia |  | Yes | 1 |
|  | Knowledge, attitude and practice towards cervical cancer among women in Finote Selam city administration, West Gojjam Zone, Amhara Region, North West Ethiopia, 2017 |  | Yes | 1 |
|  | Cervical Cancer Screening Service Utilization and Associated Factors among Women in the Shabadino District, Southern Ethiopia | Yes | | |
|  | Retrospective Review of Loop Electrosurgical Excision Procedure (LEEP) Outcomes at a Tertiary Hospital in Zambia |  | Yes | 5 |
|  | Cross-country learning networks for cervical cancer prevention in low resource settings |  | Yes | 7 |
|  | Impact of knowledge and attitude on the utilization rate of cervical cancer screening tests among Ethiopian women: A systematic review and meta-analysis |  | Yes | 5 |
|  | Hpv prevalence among iranian females |  | Yes | 7 |
|  | Utilization of cervical cancer screening services and its associated factors among primary school teachers in Ilala Municipality, Dar es Salaam, Tanzania | Yes | | |
|  | Model-based impact and cost-effectiveness of cervical cancer prevention in sub-Saharan Africa |  | Yes | 1 |
|  | Packaging health services when resources are limited: the example of a cervical cancer screening visit |  | Yes | 1 |
|  | Knowledge, attitude, and practice of cervical cancer screening among women living with HIV in the Kilimanjaro region, northern Tanzania |  | Yes | 1 |
|  | Barriers and facilitators for cervical cancer screening among adolescents and young people: a systematic review |  | Yes | 5 |
|  | Patient advocacy approaches to advancing access to care for women's cancer in East Africa |  | Yes | 7 |
|  | Approaching "precision disparities" in cervical cancer outcomes |  | Yes | 7 |
|  | Perceptions of HPV and cervical cancer among Haitian immigrant women: implications for vaccine acceptability |  | Yes | 1 |
|  | Mailed HPV self-sampling for cervical cancer screening among underserved minority women: study protocol for a randomized controlled trial |  | Yes | 1 |
|  | Burden of human papillomavirus among haitian immigrants in Miami, Florida: Community-based participatory research in action |  | Yes | 1 |
|  | Experiences and perceptions regarding clinical breast exam screening by trained laywomen in Malawi |  | Yes | 1 |
|  | Getting more than "claps": incentive preferences of voluntary community-based mobilizers in Tanzania |  | Yes | 1 |
|  | Increasing breast and cervical cancer screening uptake in women of child-bearing age in niger state, Nigeria |  | Yes | 7 |
|  | Acceptance of peer navigators to reduce barriers to cervical cancer screening and treatment among women with HIV infection in Tanzania |  | Yes | 3 |
|  | Cervical precancer treatment in Cambodia |  | Yes | 7 |
|  | Sixth year of the symposium on global cancer research: Enhancing communication and collaboration to support improved cancer prevention and control |  | Yes | 7 |
|  | Knowledge, attitudes, and practices regarding cervical cancer and screening among Ethiopian health care workers |  | Yes | 1 |
|  | Current Status and Future Perspectives of Molecular Prevention Strategies for Cervical Cancers |  | Yes | 1 |
|  | Molecular characterization of high-risk human papillomavirus (HR-HPV) in women in Lomé, Togo |  | Yes | 1 |
|  | Integration of HIV and cervical cancer screening perceptions and preferences of communities in Uganda |  | Yes | 1 |
|  | Delivering cost-effective cervical cancer screening package to women living with human immunodeficiency virus by reproductive health uganda |  | Yes | 7 |
|  | Oral contraceptives, human papillomavirus and cervical cancer |  | Yes | 1 |
|  | A Descriptive Cross-Sectional Study on Awareness and Belief of People About Cancer in Southern Ethiopia: Special Focus on Breast and Cervical Cancers |  | Yes | 1 |
|  | Sexual and reproductive health services utilization by female sex workers is context-specific: results from a cross-sectional survey in India, Kenya, Mozambique and South Africa |  | Yes | 1 |
|  | Preferences for prep delivery among FSW in Malawi using a discrete choice experiment |  | Yes | 7 |
|  | Needs for Professional Education to Optimize Cervical Cancer Screenings in Low-Income Countries: a Case Study from Tanzania |  | Yes | 1 |
|  | Exploring Complicity of Cervical Cancer Screening in Malawi: The Interplay of Behavioral, Cultural, and Societal Influences |  | Yes | 2 |
|  | Community-based via and thermocoagulation for cervical cancer prevention in rural malawi |  | Yes | 7 |
|  | Precancerous Cervical Lesions Among HIV-Infected Women Attending HIV Care and Treatment Clinics in Southwest Ethiopia: A Cross-Sectional Study |  | Yes | 1 |
|  | High prevalence of HR-HPV infection in HIV-infected women from Tanzania |  | Yes | 7 |
|  | The limitations of establishing a cervical cancer screening programme at Kabubbu Health Centre, rural Uganda |  | Yes | 7 |
|  | "A loving man has a very huge responsibility": A mixed methods study of Malawian men's knowledge and beliefs about cervical cancer |  | Yes | 1 |
|  | Cervical human papillomavirus prevalence and genotype distribution among hybrid capture 2 positive women 15 to 64 years of age in the Gurage zone, rural Ethiopia |  | Yes | 1 |
|  | Acceptability of cervical cancer screening using visual inspection among women attending a childhood immunization clinic in Uganda |  | Yes | 2 |
|  | Development and Implementation of a Culturally Appropriate Education Program to Increase Cervical Cancer Screening among Maasai Women in Rural Tanzania |  | Yes | 1 |
|  | Implementing a community-based cervical cancer screen-and-treat pilot in Malawi: Lessons learned |  | Yes | 7 |
|  | Effectiveness of One-Way Text Messaging on Attendance to Follow-Up Cervical Cancer Screening Among Human Papillomavirus-Positive Tanzanian Women (Connected2Care): Parallel-Group Randomized Controlled Trial |  | Yes | 1 |
|  | Competing needs: a qualitative study of cervical cancer screening attendance among HPV-positive women in Tanzania |  | Yes | 1 |
|  | Text messages to increase attendance to follow-up cervical cancer screening appointments among HPV-positive Tanzanian women (Connected2Care): study protocol for a randomised controlled trial |  | Yes | 5 |
|  | Barriers to attend cervical screening services in Democratic Republic of Congo |  | Yes | 7 |
|  | Prevalence and risk factors of cervical squamous intraepithelial lesions among HIV-infected women in Dar es Salaam, Tanzania |  | Yes | 1 |
|  | Cancer occurrence patterns in Maputo, Mozambique: Retrospective study from 1991 to 2008 |  | Yes | 7 |
|  | Epidemiology and prevention of human papillomavirus and cervical cancer in sub-Saharan Africa: a comprehensive review |  | Yes | 1 |
|  | Characteristics and geographic distribution of HIV-positive women diagnosed with cervical cancer in Dar es Salaam, Tanzania |  | Yes | 1 |
|  | Demographic, knowledge, attitudinal, and accessibility factors associated with uptake of cervical cancer screening among women in a rural district of Tanzania: three public policy implications |  | Yes | 2 |
|  | Knowledge towards cervical cancer prevention and screening practices among women who attended reproductive and child health clinic at Magu district hospital, Lake Zone Tanzania: a cross-sectional study |  | Yes | 1 |
|  | Feasibility and acceptability of human papillomavirus self-sampling in a semi-urban area in northern Tanzania |  | Yes | 2 |
|  | Health beliefs and practices regarding cervical cancer screening among women in Nepal: A descriptive cross‐sectional study |  | Yes | 1 |
|  | Towards eliminating cervical cancer in East Africa: Feasibility of visual inspection with acetic acid (VIA) screening and immediate cryotherapy in rural and urban Tanzania |  | Yes | 7 |
|  | Compliance with visual inspection with acetic acid (VIA) screening for cervical cancer in northern Tanzania |  | Yes | 7 |
|  | Wealth-related inequalities of women's knowledge of cervical cancer screening and service utilisation in 18 resource-constrained countries: evidence from a pooled decomposition analysis |  | Yes | 1 |
|  | Prevalence and risk factors for cervical cancer and pre-cancerous lesions in Rwanda |  | Yes | 1 |
|  | Community health workers paired with human papillomavirus self-samplers a promising method to reduce cervical cancer |  | Yes | 7 |
|  | Screening of human papillomavirus, cervical cytological abnormalities and associated risk factors in HIVpositive and HIV-negative women in Rwanda |  | Yes | 7 |
|  | Knowledge and Practices of Cervical Cancer and Its Prevention Among Malawian Women |  | Yes | 1 |
|  | Setting up a community-based cervical screening service in a low-income country: a pilot study from north-western Tanzania |  | Yes | 1 |
|  | Feasibility of self-collected HPV and thermal ablation treatment |  | Yes | 7 |
|  | Client satisfaction with cervical cancer screening in Malawi |  | Yes | 1 |
|  | Cervical cancer control and prevention in Malawi: need for policy improvement |  | Yes | 1 |
|  | Health systems challenges in cervical cancer prevention program in Malawi |  | Yes | 2 |
|  | Fighting cancer in developing countries: A cooperative model of oncology service in Uganda |  | Yes | 7 |
|  | Predictors of uptake of cervical cancer screening among women in Urban Tanzania: community-based cross-sectional study | Yes | | |
|  | Evaluation of a cervical cancer screening program based on HPV testing and LLETZ excision in a low resource setting |  | Yes | 1 |
|  | Expanding Cervical Cancer Screening and Treatment in Tanzania: Stakeholders' Perceptions of Structural Influences on Scale-Up |  | Yes | 1 |
|  | Comprehensive Cervical Cancer Prevention in Tanzania (CONCEPT) study: Cohort profile |  | Yes | 1 |
|  | HPV types, cervical high-grade lesions and risk factors for oncogenic human papillomavirus infection among 3416 Tanzanian women |  | Yes | 1 |
|  | Incidence of Breast Cancer in Eritrea: A Retrospective Study from 2011 to 2017 |  | Yes | 1 |
|  | Community cervical cancer screening: Barriers to successful home-based HPV self-sampling in Dabat district, North Gondar, Ethiopia. A qualitative study |  | Yes | 2 |
|  | Competency test for visual inspection of cervical cancer lesions with acetic acid in equatorial guinea |  | Yes | 7 |
|  | Barriers to cervical cancer screening among Europe: a scoping review immigrant women in Little Haiti, Miami |  | Yes | 5 |
|  | Cervical cancer screening "see and treat approach": real-life uptake after invitation and associated factors at health facilities in Gondar, Northwest Ethiopia. | Yes | | |
|  | Breast and cervical cancer screening among South Asian immigrants in the United States |  | Yes | 1 |
|  | HIV and predictors of advanced cancer at presentation: Uganda |  | Yes | 7 |
|  | Acceptability of HPV screening among HIV-infected women attending an HIV-dedicated clinic in Abidjan, Cote d'Ivoire |  | Yes | 1 |
|  | Precancerous Cervical Lesions and Associated Factors Among Women Attending Cervical Screening at Adama Hospital Medical College, Central Ethiopia |  | Yes | 2 |
|  | Comprehensive knowledge and uptake of cervical cancer screening is low among women living with HIV/AIDS in Northwest Ethiopia |  | Yes | 7 |
|  | Health Seeking Behavior of Patients Diagnosed with Cervical Cancer in Addis Ababa, Ethiopia |  | Yes | 1 |
|  | Community-based HPV self-collection versus visual inspection with acetic acid in Uganda: a cost-effectiveness analysis of the ASPIRE trial |  | Yes | 1 |
|  | Workshop on screening for cancer of the uterine cervix in Central America |  | Yes | 1 |
|  | Cancer incidence in the Hmong of Central California, United States, 1987-94 |  | Yes | 1 |
|  | Utilization of cervical cancer screening services and its associated factors among primary school teachers in Ilala Municipality, Dar es Salaam, Tanzania |  | Yes | 2 |
|  | Assessing women's willingness to collect their own cervical samples for HPV testing as part of the ASPIRE cervical cancer screening project in Uganda |  | Yes | 1 |
|  | Self-collection based HPV testing for cervical cancer screening among women living with HIV in Uganda: a descriptive analysis of knowledge, intentions to screen and factors associated with HPV positivity |  | Yes | 1 |
|  | Uptake of cervical cancer screening and associated factors among 15-49-year-old women in Dessie town, northeast Ethiopia |  | Yes | 7 |
|  | Quality assurance and risk reduction guidelines |  | Yes | 1 |
|  | Effect of an Intervention in General Practice to Increase the Participation of Immigrants in Cervical Cancer Screening: A Cluster Randomized Clinical Trial |  | Yes | 1 |
|  | Quality assurance and risk reduction guidelines |  | Yes | 1 |
|  | [Diagnosis and treatment of invasive cervical cancer in Cambodia (apropos of 35 cases)] |  | Yes | 1 |
|  | Hpv vaccination, making a difference in global cancer incidence rates |  | Yes | 7 |
|  | [Prevention of cervical cancer (II): prophylactic HPV vaccination, current knowledge, practical procedures and new issues] |  | Yes | 1 |
|  | Implementation of cervical cancer screening using visual inspection with acetic acid in rural Mozambique: successes and challenges using HIV care and treatment programme investments in Zambézia Province |  | Yes | 1 |
|  | Cancer epidemiology in the pacific islands - past, present and future |  | Yes | 1 |
|  | Cervical cancer in the asian pacific-epidemiology, screening and treatment |  | Yes | 1 |
|  | Supporting radiation oncology in south East Asia: An overview |  | Yes | 7 |
|  | Cervical Cancer Awareness among Women in Tanzania: An Analysis of Data from the 2011-12 Tanzania HIV and Malaria Indicators Survey |  | Yes | 1 |
|  | "When You Have Gotten Help, That Means You Were Strong": A Qualitative Study of Experiences in a "Screen and Treat" Program for Cervical Cancer Prevention in Malawi |  | Yes | 1 |
|  | Health care workers' experiences with implementation of "screen and treat" for cervical cancer prevention in Malawi: A qualitative study |  | Yes | 1 |
|  | Cervical cancer knowledge and attitudes among HIV-positive men in Malawi |  | Yes | 7 |
|  | Cervical cancer knowledge and perceptions among women in Malawi: Qualitative data from a high-burden, low-resource setting |  | Yes | 1 |
|  | [Precancerous lesions of the uterine cervix in Pointe-Noire, Congo] |  | Yes | 1 |
|  | Determinants of visiting a referral hospital for cervical cancer screening at Uganda Cancer Institute |  | Yes | 7 |
|  | Cervical cancer screening uptake and challenges in Malawi from 2011 to 2015: retrospective cohort study |  | Yes | 5 |
|  | Knowledge and practices about cervical cancer and screening among married men in traditional authority nkhumba, phalombe district |  | Yes | 5 |
|  | International Image Concordance Study to Compare a Point-of-Care Tampon Colposcope With a Standard-of-Care Colposcope |  | Yes | 1 |
|  | Factors influencing the uptake of cervical cancer screening services in Tanzania: A health system perspective from national and district levels |  | Yes | 2 |
|  | Implementation of Cervical cancer screening services at 9 IHV/PEPFAR supported ART clinics in Uganda; Successes and challenges |  | Yes | 7 |
|  | Women's knowledge and attitudes towards cervical cancer prevention: a cross sectional study in Eastern Uganda |  | Yes | 1 |
|  | Persistence rate of cervical human papillomavirus infections and abnormal cytology in Rwanda |  | Yes | 1 |
|  | Screening for human papillomavirus, cervical cytological abnormalities and associated risk factors in HIV-positive and HIV-negative women in Rwanda |  | Yes | 1 |
|  | Predictors of cervical cancer screening service utilization among commercial sex workers in Northwest Ethiopia: a case-control study |  | Yes | 1 |
|  | HIV status, age at cervical Cancer screening and cervical cytology outcomes in an opportunistic screening setting in Nigeria: a 10-year Cross sectional data analysis |  | Yes | 1 |
|  | HIV and development of epithelial cell abnormalities in women with prior normal cervical cytology in Nigeria |  | Yes | 1 |
|  | Cervical cancer survival in a resource-limited setting-North Central Nigeria |  | Yes | 1 |
|  | Protocol for the study of cervical cancer screening technologies in HIV-infected women living in Rwanda |  | Yes | 5 |
|  | Improving access to care through community engagement: Zambian case |  | Yes | 7 |
|  | Efficacy of antiviral drug AV2 in the treatment of human papillomavirus-associated precancerous lesions of the uterine cervix: A randomized placebo-controlled clinical trial in Kinshasa, DR Congo. (KINVAV study) |  | Yes | 1 |
|  | Difficulties in implementing a HPV/cervical cancer screening programme in Kinshasa |  | Yes | 7 |
|  | Influences on uptake of reproductive health services in Nsangi community of Uganda and their implications for cervical cancer screening |  | Yes | 1 |
|  | Knowledge, attitudes and practices on cervical cancer screening among the medical workers of Mulago Hospital, Uganda |  | Yes | 1 |
|  | Awareness of cervical cancer risk factors and symptoms: cross-sectional community survey in post-conflict northern Uganda |  | Yes | 1 |
|  | Mind the gaps: a qualitative study of perceptions of healthcare professionals on challenges and proposed remedies for cervical cancer help-seeking in post conflict northern Uganda |  | Yes | 1 |
|  | Cervical cytological changes in HIV-infected patients attending care and treatment clinic at Muhimbili National Hospital, Dar es Salaam, Tanzania |  | Yes | 2 |
|  | Utilization of cervical cancer screening services and trends in screening positivity rates in a 'screen-and-treat' program integrated with HIV/AIDS care in Zambia |  | Yes | 1 |
|  | OncoE6 Positivity among VIA Positive Suspected Cases from Colposcopy Clinic of Mymensingh Medical College Hospital, Mymensingh |  | Yes | 1 |
|  | Molecular Diagnosis of Human Papilloma Virus by PCR |  | Yes | 1 |
|  | A public health approach to cervical cancer screening in Africa through community-based self-administered HPV testing and mobile treatment provision |  | Yes | 1 |
|  | A report on the Marrakech International Women's Cancer Days: dialogs and implications |  | Yes | 1 |
|  | Uptake of Cervical Cancer Screening and Associated Factors among Women in Rural Uganda: A Cross Sectional Study | Yes | | |
|  | Knowledge, facilitators and barriers to cervical cancer screening among women in Uganda: a qualitative study |  | Yes | 2 |
|  | Knowledge and practices of general practitioners at district hospitals towards cervical cancer prevention in Burundi, 2015: a cross-sectional study |  | Yes | 1 |
|  | Prevalence and genotype-specific distribution of human papillomavirus in Burundi according to HIV status and urban or rural residence and its implications for control |  | Yes | 1 |
|  | Knowledge and practices of general practitioners at district hospitals towards cervical cancer prevention in Burundi, 2015: a cross-sectional study |  | Yes | 1 |
|  | Caribbean cancer control leadership forums |  | Yes | 7 |
|  | Low uptake of cervical cancer screening among HIV positive women in Gondar University referral hospital, Northwest Ethiopia: cross-sectional study design |  | Yes | 1 |
|  | Cost-Effectiveness of Screening and Treatment for Cervical Cancer in Tanzania: Implications for other Sub-Saharan African Countries |  | Yes | 1 |
|  | Comparison between visual inspection of cervix and cytology based screening procedures in Bangladesh |  | Yes | 1 |
|  | Role of the HPV DNA test in follow-up of treated cervical intraepithelial neoplasia in Bangladesh |  | Yes | 1 |
|  | Situational analysis for diagnosis and treatment of cervical cancer in mainland Tanzania |  | Yes | 1 |
|  | Abnormal cervical lesions and its associated factors using visual inspection with acetic acid (VIA) at a referral hospital in ethiopia |  | Yes | 7 |
|  | Cervical cancer screening service utilization and associated factors among age-eligible women in Jimma town using health belief model, South Cervical cancer screening service utilization and associated factors among age-eligible women in Jimma town using health belief model, South West Ethiopia | Yes | | |
|  | Knowledge Toward Cervical Cancer and Its Determinants Among Women Aged 30-49 in Jimma Town, Southwest Ethiopia |  | Yes | 2 |
|  | Cervical cancer prevention in Nepal through health system strengthening from rural screening camps to tertiary level care: A collaborative approach |  | Yes | 7 |
|  | Cervical Cancer Screening, Adherence to and Challenges of Follow-Up in Resources Poor Setting |  | Yes | 2 |
|  | Using Film to Disseminate Information on Cervical Cancer Prevention in Lusaka: Results from a Small Intervention Study |  | Yes | 1 |
|  | Differences in Cervical Cancer Screening Knowledge and Practices by HIV Status and Geographic Location: Implication for Program Implementation in Zambia |  | Yes | 1 |
|  | Associations between methylation of paternally expressed gene 3 (PEG3), cervical intraepithelial neoplasia and invasive cervical cancer |  | Yes | 1 |
|  | Knowledge, attitudes, and practice of cervical cancer prevention among health workers in rural health centres of Northern Uganda |  | Yes | 1 |
|  | Factors associated with cervical cancer screening participation among migrant women in Europe: a scoping review |  | Yes | 5 |
|  | Prevalence and severity of cervical squamous intraepithelial lesion in a tertiary hospital in northern Tanzania |  | Yes | 1 |
|  | Innovative public-private partnership: a diagonal approach to combating women's cancers in Africa |  | Yes | 1 |
|  | Factors Associated with Uptake of Visual Inspection with Acetic Acid (VIA) for Cervical Cancer Screening in Western Kenya |  | Yes | 2 |
|  | Role of gender in perspectives of discrimination, stigma, and attitudes relative to cervical cancer in rural Senegal |  | Yes | 1 |
|  | Role of gender in perspectives of discrimination, stigma, and attitudes relative to cervical cancer in rural Sénégal |  | Yes | 1 |
|  | Public health implications of HPV and HSV vaccinations |  | Yes | 7 |
|  | Expanding the Single-Visit Approach for Cervical Cancer Prevention: Successes and Lessons From Burkina Faso |  | Yes | 1 |
|  | Factors influencing breast and cervical cancer screening service delivery in Malawi: A systematic review |  | Yes | 7 |
|  | eC3--a modern telecommunications matrix for cervical cancer prevention in Zambia |  | Yes | 1 |
|  | Effectiveness of a program to prevent cervical cancer among HIV-infected women in Zambia |  | Yes | 7 |
|  | Prevalence and predictors of squamous intraepithelial lesions of the cervix in HIV-infected women in Lusaka, Zambia |  | Yes | 1 |
|  | Screen-and-Treat Approach to Cervical Cancer Prevention Using Visual Inspection With Acetic Acid and Cryotherapy : Experiences, Perceptions, and Beliefs From Demonstration Projects in Peru, Uganda, and Vietnam |  | Yes | 1 |
|  | [Infiltrating carcinoma of the uterine cervix: epidemiology, prognostic factors, therapeutic strategies] |  | Yes | 1 |
|  | Promoters of and barriers to cervical cancer screening in a rural setting in Tanzania | Yes | | |
|  | Incidence and risk factors for cervical cancer and pre-cancerous lesions in Equatorial Guinea |  | Yes | 7 |
|  | Integrating reproductive health services into HIV care: Strategies for successful implementation in a lowresource HIV clinic in Lilongwe, Malawi |  | Yes | 1 |
|  | Women's perception of cervical cancer and its prevention in rural Laos |  | Yes | 1 |
|  | A hands-on training course for cervical cancer screening and management of pre-invasive disease in Lesotho, Africa |  | Yes | 7 |
|  | Integrating HIV testing into cervical cancer screening in Tanzania: an analysis of routine service delivery statistics |  | Yes | 1 |
|  | cAREHPVTM experience in four countries |  | Yes | 7 |
|  | Implementation and feasibility of an adapted two-stage visual inspection with acetic acid/cryotherapy-based cervical cancer screening programme for HIV-infected women in Addis Ababa, Ethiopia |  | Yes | 1 |
|  | An epidemiologic study of lifestyle of cancer patients presenting at National Hospital and Cancer Research Centre Pvt. Ltd., Jawalakhel, Nepal |  | Yes | 7 |
|  | A Mobile Health Data Collection System for Remote Areas to Monitor Women Participating in a Cervical Cancer Screening Campaign |  | Yes | 1 |
|  | A community-based intervention to increase participation in cervical cancer screening among immigrants in Norway |  | Yes | 1 |
|  | What do healthcare providers know about human papillomavirus (HPV) and cervical cancer? a cross-sectional knowledge assessment in debre markos, Ethiopia |  | Yes | 7 |
|  | Knowledge regarding cervical cancer among undergraduate female students at a selected college of Lalitpur, Nepal |  | Yes | 1 |
|  | Cervical cancer screening, barriers and propositions to make screening more accessible in India |  | Yes | 7 |
|  | Awareness, practice and barriers to nonparticipation in cervical cancer screening among Sikkimese women in India |  | Yes | 7 |
|  | Cervical cancer screening decentralized policy adaptation: an African rural-context-specific systematic literature review |  | Yes | 1 |
|  | Determinants of Cervical Cancer Screening Accuracy for Visual Inspection with Acetic Acid (VIA) and Lugol's Iodine (VILI) Performed by Nurse and Physician |  | Yes | 1 |
|  | Association of knowledge, attitude and demographic variables with cervical Pap smear practice in Nepal |  | Yes | 1 |
|  | Awareness and prevalence of cervical cancer screening among women in Nepal |  | Yes | 1 |
|  | A Community Health Education System to meet the health needs of Indo-Chinese women |  | Yes | 1 |
|  | Acceptability and preferences for self-collected screening for cervical cancer within health systems in rural Uganda: A mixed-methods approach |  | Yes | 1 |
|  | Current status of knowledge, attitude and practice (KAP) and screening for cervical cancer in countries at different levels of development |  | Yes | 2 |
|  | Culturally informed views on cancer screening: a qualitative research study of the differences between older and younger Somali immigrant women |  | Yes | 1 |
|  | The knowledge, attitude and practice regarding the cervical cancer prevention among final year undergraduate nurse and midwife students in antananarivo, madagascar |  | Yes | 7 |
|  | Before you teach me, I cannot know': immigrant women's barriers and enablers with regard to cervical cancer screening among different ethnolinguistic groups in Canada |  | Yes | 1 |
|  | Knowledge and attitudes regarding routine health screening and prevention in Somali, Vietnamese, and Latina women |  | Yes | 1 |
|  | Reaching unreached population: Advancing cervical cancer prevention in low-middle income countries |  | Yes | 7 |
|  | Pap Testing in a High-Income Country with Suboptimal Compliance Levels: A Survey on Acceptance Factors among Sicilian Pap Testing in a High-Income Country with Suboptimal Compliance Levels: A Survey on Acceptance Factors among Sicilian Women |  | Yes | 1 |
|  | Diagnostic value of papanicolaou cytology when human papillomavirus status is known |  | Yes | 7 |
|  | Cervical cancer prevention in rural Haiti: The experience of one health service organisation |  | Yes | 7 |
|  | Management of locally advanced cervical cancer |  | Yes | 1 |
|  | Factors related to cervical cancer screening differences between regions around Bagamoyo, Tanzania |  | Yes | 7 |
|  | Prevalence and predictors of a positive cervical cancer screening test in a sexually transmitted infection clinic in Lilongwe, Malawi |  | Yes | 7 |
|  | Cervical cancer screening in rural Ethiopia: a cross- sectional knowledge, attitude and practice study |  | Yes | 1 |
|  | Cervical cancer in Tanzania: A systematic review of current challenges in six domains |  | Yes | 5 |
|  | Cervical Cancer Prevention-Cervical Screening: Science in Evolution |  | Yes | 1 |
|  | Difficulty with single-visit approach (SVA) for colposcopy and cervical intraepithelial neoplasia (CIN) treatment in Goma/RDC |  | Yes | 7 |
|  | Cervical cancer: a missed health priority in Tanzania |  | Yes | 1 |
|  | Implementing cervical cancer prevention programs at national scale in developing countries: Mozambique's successful experience |  | Yes | 7 |
|  | Overview of cervical cancer screening practices in the extended Middle East and North Africa countries |  | Yes | 1 |
|  | Human papillomavirus infection and cervical cancer prevention in India, Bangladesh, Sri Lanka and Nepal |  | Yes | 1 |
|  | The challenge of AIDS-related malignancies in sub-Saharan Africa |  | Yes | 1 |
|  | Cervical Cancer Screening Behavior among Nepalese Women |  | Yes | 1 |
|  | Prevalence and correlates of mycoplasma genitalium in HIV-positive African women |  | Yes | 7 |
|  | Screening for cervical cancer in Butha Buthe, Lesotho. A study of Papanicolaou (Pap) smears in a previously unscreened community over a one-year period |  | Yes | 1 |
|  | Addressing barriers to cervical cancer screening in rural Uganda |  | Yes | 7 |
|  | Cancer Screening Utilization Among Immigrant Women in Miami, Florida |  | Yes | 1 |
|  | Intravaginal practices are associated with greater odds of high-risk HPV infection in Haitian women |  | Yes | 1 |
|  | Impact of Initiating Screening Programs on Referral and Management of Cervical Cancer in Tanzania |  | Yes | 1 |
|  | Disease prevalence, tumour stage, and results of testing in the pilot phase of a service for cervical cancer screening and diagnosis in northern Tanzania |  | Yes | 7 |
|  | Community-Based Health Education has Positive Influence on the Attitude to Cervical Cancer Screening among Women in Rural Nepal |  | Yes | 1 |
|  | Prevalence of human papillomavirus infection among women in rural Nepal |  | Yes | 1 |
|  | The Single-Visit Approach as a Cervical Cancer Prevention Strategy Among Women With HIV in Ethiopia: Successes and Lessons Learned |  | Yes | 1 |
|  | Knowledge about cervical cancer and barriers toward cervical cancer screening among HIV-positive women attending public health centers in Addis Ababa city, Ethiopia |  | Yes | 3 |
|  | Risk factors for cervical cancer in low resource country |  | Yes | 7 |
|  | Awareness of cervical cancer and screening among rural Nepalese women |  | Yes | 7 |
|  | Breast cancer knowledge and screening practice among women visited to KIST medical college |  | Yes | 1 |
|  | Prevalence and risk factors for High-Risk Human Papillomavirus (hrHPV) infection among HIV-infected and Uninfected Rwandan women: implications for hrHPV-based screening in Rwanda |  | Yes | 1 |
|  | Knowledge, attitudes and practices of cervical cancer screening among rural and urban women in Kilimanjaro, Tanzania |  | Yes | 7 |
|  | Predictors of cervical cancer screening practice among HIV positive women attending adult anti-retroviral treatment clinics in Bishoftu town, Ethiopia: the application of a health belief model |  | Yes | 3 |
|  | [Pap smear for mass screening: Results of an African experiment] |  | Yes | 1 |
|  | Cervical cancer: What vaccine in Senegal? |  | Yes | 7 |
|  | HPV-Chlamydial Coinfection, Prevalence, and Association with Cervical Intraepithelial Lesions: A Pilot Study at Mbarara Regional Referral Hospital |  | Yes | 1 |
|  | Patient Preferences and Willingness to Pay for Cervical Cancer Prevention in Zambia: Protocol for a Multi-Cohort Discrete Choice Experiment |  | Yes | 1 |
|  | HPV and cervical cancer: updates on an established relationship |  | Yes | 1 |
|  | HPV positivity among women in Central Uganda participating in a community health campaign offering self-collected HPV-based cervical cancer screening |  | Yes | 7 |
|  | Surgical candidacy and treatment initiation among women with cervical cancer at public referral hospitals in Kampala, Uganda: a descriptive cohort study |  | Yes | 1 |
|  | Evidence-based improvisation: Facing the challenges of cervical cancer care in Uganda |  | Yes | 1 |
|  | Improving uptake of cervical cancer screening services for women living with HIV and attending chronic care services in rural Malawi |  | Yes | 1 |
|  | Health professionals' willingness to pay and associated factors for cervical cancer screening program at College of Medicine and Health Sciences, University of Gondar, Northwest Ethiopia |  | Yes | 2 |
|  | Barriers and facilitators to cervical cancer screening in Nepal: A qualitative study | Yes | | |
|  | Risk factors of precancerous cervical lesions: The role of women's socio-demographic, sexual behavior and body mass index in Amhara region referral hospitals; case-control study |  | Yes | 1 |
|  | Factors affecting utilization of cervical cancer screening services among women attending public hospitals in Tigray region, Ethiopia, 2018; Case control study |  | Yes | 5 |
|  | Knowledge, attitudes and practices among Brazzaville midwives on cervical cancer screening |  | Yes | 1 |
|  | Hiv testing and counseling services among confirmed cases of invasive cervical cancer patients at Tikur Anbesa Specialized Teaching Hospital |  | Yes | 7 |
|  | Uptake of Cervical Cancer Screening and Associated Factors Among 15-49-Year-Old Women in Dessie Town, Northeast Ethiopia |  | Yes | 1 |
|  | Cervical cancer screening in Mali: Eight years experience and perspectives |  | Yes | 7 |
|  | Determinants of VIA Positivity Among Women Screened for Cervical Precancerous Lesion in Public Hospitals of Oromia Region, Ethiopia: Unmatched Case-Control Study |  | Yes | 1 |
|  | Knowledge, Attitude and Practice Towards Cervical Cancer Screening Among Women and Associated Factors in Hospitals of Wolaita Zone, Southern Ethiopia |  | Yes | 1 |
|  | Prevalence of oncogenic human papillomavirus (HPV 16/18) infection, cervical lesions and its associated factors among women aged 21-49 years in Amhara region, Northern Ethiopia |  | Yes | 1 |
|  | Understanding the role of embarrassment in gynaecological screening: a qualitative study from the ASPIRE cervical cancer screening project in Uganda |  | Yes | 1 |
|  | Human papillomavirus (HPV) infection, HIV infection and cervical cancer in Tanzania, east Africa |  | Yes | 1 |
|  | Breast cancer clinicopathological presentation, gravity and challenges in Eritrea, East Africa: management practice in a resource-poor setting |  | Yes | 1 |
|  | Liquid-based cytology for the detection of cervical intraepithelial lesions in Jimma town, Ethiopia |  | Yes | 1 |
|  | Cervical Cancer Awareness and Practice of Pap Smear Test Among Women with Gynecological problems |  | Yes | 1 |
|  | Knowledge, attitude, practice and barriers of cervical cancer screening among women living in mid-western rural, Nepal | Yes | | |
|  | Prospective cohort study examining cervical cancer screening methods in HIV-positive and HIV-negative Cambodian Women: a comparison of human papilloma virus testing, visualization with acetic acid and digital colposcopy |  | Yes | 1 |
|  | Prevalence of Cervical Dysplasia in HIV-Positive and HIV-Negative Women at the Sihanouk Hospital Center of HOPE, Phnom Penh , Cambodia |  | Yes | 1 |
|  | Contribution of the burkinabe society of obstetrics and gynecology (SOGOB) to cervical cancer prevention in Burkina Faso |  | Yes | 7 |
|  | Visual inspection with acetic acid followed by cryotherapy, a single visit approach to cervical cancer prevention |  | Yes | 7 |
|  | Using Internet-platform technology to traincommunity-based researchers in developing countries:Lessons Learned from a Caribbean study |  | Yes | 7 |
|  | Knowledge, attitude and practice of cervical cancer screening and associated factors amongst female students at Wollega University, western Ethiopia |  | Yes | 6 |
|  | Raising awareness about women's cancer and increasing local engagement with healthcare services in Haiti |  | Yes | 7 |
|  | Screening for human papillomavirus, cervical cytological abnormalities and associated risk factors in HIV‐positive and HIV‐negative women in Rwanda |  | Yes | 1 |
|  | Knowledge, attitudes, and practices toward cervical cancer prevention among women in Kampong Speu Province, Cambodia |  | Yes | 1 |
|  | Epidemiology of abnormal cervical cytology in female sex workers in Mali, West Africa |  | Yes | 7 |
|  | Human papillomavirus genotype distribution and factors associated among female Human papillomavirus genotype distribution and factors associated among female sex workers in West Africa |  | Yes | 2 |
|  | Prevalence and Factors Associated With HIV and Sexually Transmitted Infections Among Female Sex Workers in Bamako, Mali |  | Yes | 2 |
|  | [Cytological detection of lesions of the uterine cervix: results of a campaign in Senegal] |  | Yes | 1 |
|  | [Cervical cancer in developing countries. A threat to reproductive health] |  | Yes | 1 |
|  | Knowledge, Attitude, and Practice on Cervical Cancer Screening and Associated Factors Among Women Aged 15-49 Years in Adigrat Town, Northern Ethiopia, 2019: A Community-Based Cross-Sectional Study |  | Yes | 1 |
|  | Overcoming barriers and ensuring access to HPV vaccines in low-income countries |  | Yes | 2 |
|  | The landscape of cervical precancer treatment in low-resource settings: Not a pretty picture |  | Yes | 7 |
|  | HPV vaccination: What practical experience tells us to date |  | Yes | 7 |
|  | Opportunities and challenges for introducing HPV testing for cervical cancer screening in sub-Saharan Africa |  | Yes | 1 |
|  | Understanding the Low Level of Cervical Cancer Screening in Masaka Uganda Using the ASE Model: A Community-Based Survey |  | Yes | 1 |
|  | Worldwide trends in cervical cancer incidence: impact of screening against changes in disease risk factors |  | Yes | 1 |
|  | Social Responsibility in Cancer Prevention Research: IARC as a 'Global Science Force' |  | Yes | 1 |
|  | The health minister's response to managing cervical cancer in low-income countries |  | Yes | 7 |
|  | [Immigration from countries with a strong migratory pressure and participation in cervical cancer screening program in the Local Health Unit 2, Umbria Region. Impact on the probability of high-grade lesions and cervical cancer] |  | Yes | 1 |
|  | Distribution of HPV genotypes in cervical intraepithelial lesions and cervical cancer in Tanzanian women |  | Yes | 1 |
|  | A review of screening strategies for cervical cancer in Bahir Dar Town, virus-positive women in sub-Saharan Africa |  | Yes | 3 |
|  | Prevalence of Abnormal Cervical Lesions and Associated Factors Among Women in Harar, Eastern Ethiopia |  | Yes | 1 |
|  | Survival status and associated factors of death among cervical cancer patients attending at Tikur Anbesa Specialized Hospital, Addis Ababa, Ethiopia: a retrospective cohort study |  | Yes | 1 |
|  | Cervical Cancer Screening in the United States-Affiliated Pacific Islands: Options and Opportunities |  | Yes | 1 |
|  | Women's knowledge of and attitudes toward cervical cancer and cervical cancer screening in Zanzibar, Tanzania: a cross-sectional study |  | Yes | 1 |
|  | Motivations and experiences of women who accessed "see and treat" cervical cancer prevention services in Zambia |  | Yes | 1 |
|  | Factors associated with extensive cervical lesions among HIV-infected women screening for aids clinical trials group (actg) protocol A5282 |  | Yes | 7 |
|  | Social determinants of health associated with cervical cancer screening among women living in developing countries: a scoping review |  | Yes | 5 |
|  | Socio-demographic characteristics and associated factors influencing cervical cancer screening among women attending in St. Paul's Teaching and Referral Hospital, Ethiopia | Yes | | |
|  | Comprehensive Knowledge towards Cervical Cancer and Associated Factors among Women in Durame Town, Southern Ethiopia |  | Yes | 2 |
|  | Determining behavioral intention and its predictors towards cervical cancer screening among women in Gomma district, Jimma, Ethiopia: Application of the theory of planned behavior |  | Yes | 2 |
|  | A community health worker-led multimedia intervention to increase cervical cancer screening uptake among South Asian women: study protocol for a cluster randomized wait-list controlled trial |  | Yes | 1 |
|  | Evaluation of a Smartphone-Based Training Strategy Among Health Care Workers Screening for Cervical Cancer in Northern Tanzania: The Kilimanjaro Method |  | Yes | 1 |
|  | The use of an mHealth strategy to detect and treat cervical cancer in Tanzania |  | Yes | 7 |
|  | Smartphone-Enhanced Training, QA, Monitoring, and Evaluation of a Platform for Secondary Prevention of Cervical Cancer: Opportunities and Challenges to Implementation in Tanzania |  | Yes | 1 |
|  | Hitting two birds with one stone: Provider initiaMulanjeted counseling and testing for HIV and cervical cancer |  | Yes | 7 |
|  | Scaling up comprehensive cervical cancer prevention as a national priority of the government of Tanzania |  | Yes | 7 |
|  | Cervical cancer in the developing world |  | Yes | 1 |
|  | Factors Associated with Delayed Diagnosis of Cervical Cancer in Tikur Anbesa Specialized Hospital, Ethiopia, 2019: Cross-Sectional Study |  | Yes | 7 |
|  | Elevated IGF2 mRNA binding protein 3 (IGF2BP3) expression is associated with high-grade intraepithelial lesion in cervical brushed cells |  | Yes | 1 |
|  | Factors associated with cervical cancer screening among women aged 25-60 years in Lao People's Democratic Republic | Yes | | |
|  | Knowledge and practice of cervical cancer screening and associated factors among reproductive age group women in districts of Gurage zone, Southern Ethiopia. A cross-sectional stud | Yes | | |
|  | Knowledge on cervical cancer, attitude toward its screening, and associated factors among reproductive age women in Metu Town, Ilu Aba Bor, South West Ethiopia, 2018: community-based cross-sectional study | Yes | | |
